# Mixing Functionality in Polymer Electrolytes: A New Horizon for Achieving High‐Performance All‐Solid‐State Lithium Metal Batteries

**DOI:** 10.1002/anie.202422169

**Published:** 2025-02-28

**Authors:** Yufeng Ren, Suli Chen, Mateusz Odziomek, Junhong Guo, Pengwu Xu, Haijiao Xie, Zhihong Tian, Markus Antonietti, Tianxi Liu

**Affiliations:** ^1^ The Key Laboratory of Synthetic and Biological Colloids Ministry of Education School of Chemical and Material Engineering Jiangnan University Wuxi 214122 P. R. China; ^2^ Hangzhou Yanqu Information Technology Co. Ltd. Hangzhou 310003 P. R. China; ^3^ Engineering Research Center for Nanomaterials Henan University Kaifeng 475004 P. R. China; ^4^ Department of Colloid Chemistry Max Planck Institute of Colloids and Interfaces Am Mühlenberg 1 Potsdam 14476 Germany

**Keywords:** All-Solid-State Lithium Metal Batteries, Polymer Electrolytes, Topological Cross-linking, Ionic Migration Kinetics, Mechanical properties

## Abstract

Solid polymer electrolytes (SPEs) are a key materials component for all‐solid‐state lithium metal batteries (ASSLMBs). In these membrane‐like films, accelerating Li^+^ migration while enhancing the mechanical strength of SPEs is challenging. Herein, we introduce a new concept of supramolecularly organized, cross‐linked polymer electrolyte (PCPE) by mixing an ion‐conducting, multi‐arm boron‐containing oligomer (MBO) solid plasticizer into a polyethylene oxide (PEO)‐lithium salt matrix. Studies reveal that the Lewis acid‐base interaction between the Lewis‐acidic boron sites of MBO and lithium‐salt anions induces an amorphous MBO‐salt assembly subphase with percolating nanochannels for rapid Li^+^ transport. Meanwhile, due to the structural compatibility of the multiple linear arms of MBO with PEO, a supramolecular polymer network is obtained which partly crystallizes around the ionic nanodomains, offering an PCPE with improved mechanical strength hosting interconnected ion transport pathways. The resulting PCPE shows simultaneously enhanced ionic conductivity, improved mechanical properties, and film interface stability, which enable a dendrite‐free Li/Li symmetric cell which could be cycled over 2600 h. Excellent electrochemical performance is also demonstrated in a close‐to‐practical high capacity ASSLMBs.

## Graphical abstract

We demonstrated a supramolecularly extended/cross‐linked polymer electrolyte (PCPE) by introducing an elaborate MBO plasticizer in polymer matrix for ASSLMBs. This innovative PCPE system possesses unique topological cross‐linking structure and Lewis‐acid interaction driven MBO‐salt assembly superstructure, effectively overcomes the discrepancy between accelerating Li^+^ migration kinetics and enhancing mechanical robustness, thus achieving excellent electrochemical performances of practical ASSLMBs.

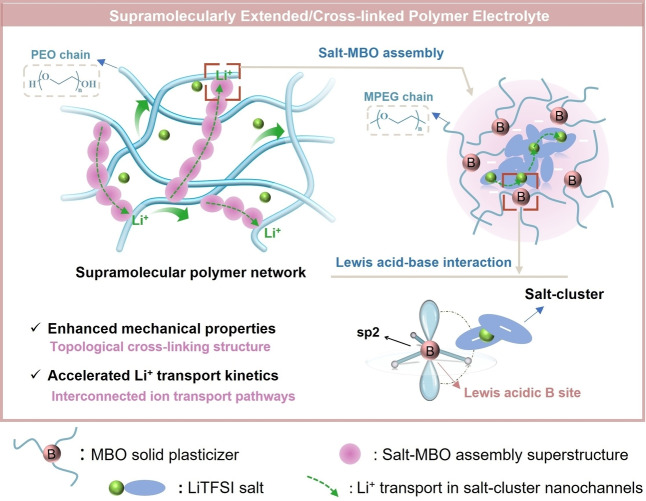

## Introduction

Lithium metal batteries (LMBs) have significantly contributed to the development of portable electronics, electric vehicles, and grid energy storage systems because of the high theoretical specific capacity (3860 mAh g^−1^) and low negative potential (**−**3.04 V vs. the standard hydrogen electrode) of metallic lithium.[^1^, ^2^, ^3^, ^4^] Unfortunately, LMBs using liquid electrolytes suffer from severe safety hazards due to the disadvantages of conventional liquid electrolytes, including their inherent volatility, flammability, thermal instability, and liquid leakage. Moreover, liquid electrolytes often exhibit chemical/electrochemical instability with metallic lithium, which will lead to unstable solid electrolyte interphase (SEI) layers and uncontrollable lithium dendrites, while the large volume changes in the lithium metal would cause localized pressure accumulation.[^5^, ^6^, ^7^] All these safety issues have severely restricted practical applications of high energy density LMBs.

Replacing liquid electrolytes by intrinsically safe solid‐state electrolytes for all‐solid‐state lithium metal batteries (ASSLMBs) is considered the most promising approach to the next‐generation batteries, which must combine large energy density and high safety.[^8^, ^9^] Thus, various solid‐state electrolytes have been applied to construct the ASSLMBs. Among these, solid polymer electrolytes (SPEs) composing of a polymer matrix and lithium salts, have been paid much attention because of the advantages of high flexibility, ease processing, and good electrode/electrolyte contact.[^10^, ^11^] However, current SPEs suffer from low ionic conductivity and inferior mechanical properties, which at the moment prohibit their broader application.[^12^, ^13^, ^14^] Based on the Li^+^ conduction mechanism of SPEs, Li^+^ migration is realized by complexation and decomplexation with polar groups in polymer chains, and thereby enabled by segmental motion of the polymer chains.[^7^, ^15^] The lower glass is the transition temperature of polymer mixture, the stronger are the segmental motions and the more favorable is Li^+^ transport.[^16^, ^17^] During the last decade, numerous publications on SPEs have focused on optimizing the amount and the mobility of the amorphous region in the polymer by (i) introducing organic plasticizers and (ii) changing the polymer topology to suppress crystallization, e.g. by constructing a cross‐linked polymer network.[^18^, ^19^] The crosslinking to networks can also improve the mechanical strength of SPEs, but the introduced rigidity again limits segmental motion and the coupled ionic conductivity.[^20^, ^21^] Introducing high boiling, noncombustible organic plasticizers, such as succinonitrile (SN) or ionic liquids, into SPEs can significantly increase the volume fraction of the amorphous subphase and promotes a more rapid Li^+^ migration, but inevitably deteriorates the mechanical properties (Figure S1).[^22^, ^23^, ^24^, ^25^, ^26^] Despite numerous studies, it is still challenging to realize a combination of high ionic conductivity and good mechanical properties of SPEs.

**Figure 1:**
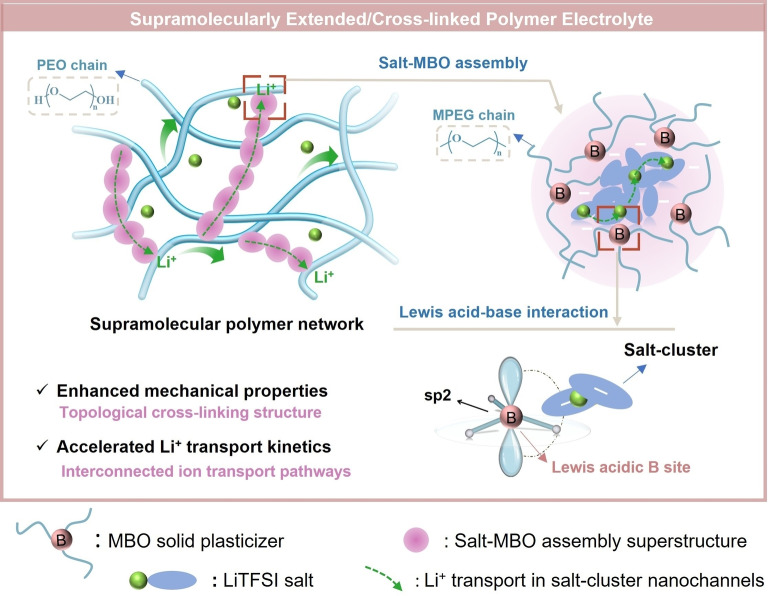
Schematic illustration of rational design of the PCPE at molecular level.

Herein, we report a supramolecularly reinforced/cross‐linked polymer electrolyte (PCPE) for ASSLMBs by introducing a multi‐arm boron‐containing oligomer (MBO) of various molecular weight into high molecular weight polyethylene oxide (PEO)‐lithium salt matrix. We show that this strategy enables the simultaneous enhancement of ionic conductivity and mechanical properties (Figure 1). The MBO was composed of Lewis‐acidic boron core and three linear methoxy‐terminated polyethylene glycol (MPEG) oligomeric arms and acts as multifunctional solid plasticizer decreasing both the glass transitions and the crystallinity of PEO. The Lewis acid properties of boron induce the formation of an unique MBO‐salt assembly superstructure to create interconnected nanochannels for rapid Li^+^ percolation through supramolecular interactions and structure formation, leading to facilitated ionic conductivity with improved Li^+^ transference number. Benefiting from the advantages of multiple linear MPEG arms of MBO (e.g. high flexibility with high segmental motion at it provides three chain ends) and the structural compatibility with PEO, a low‐T_g_ supramolecular extended polymer network is obtained. Here, an increase of physical cross‐links either by ion interaction or by altered crystallization texture can be quantified by rheology measurements. With these advantages, the PCPE exhibits simultaneously enhanced electrochemical performance and mechanical properties. Both Li/Li symmetric cells and ASSLMBs are assembled and characterized, and the results demonstrate the high application potential of the PCPE.

## Results and Discussion

### MBO Solid Plasticizer

**Figure 2:**
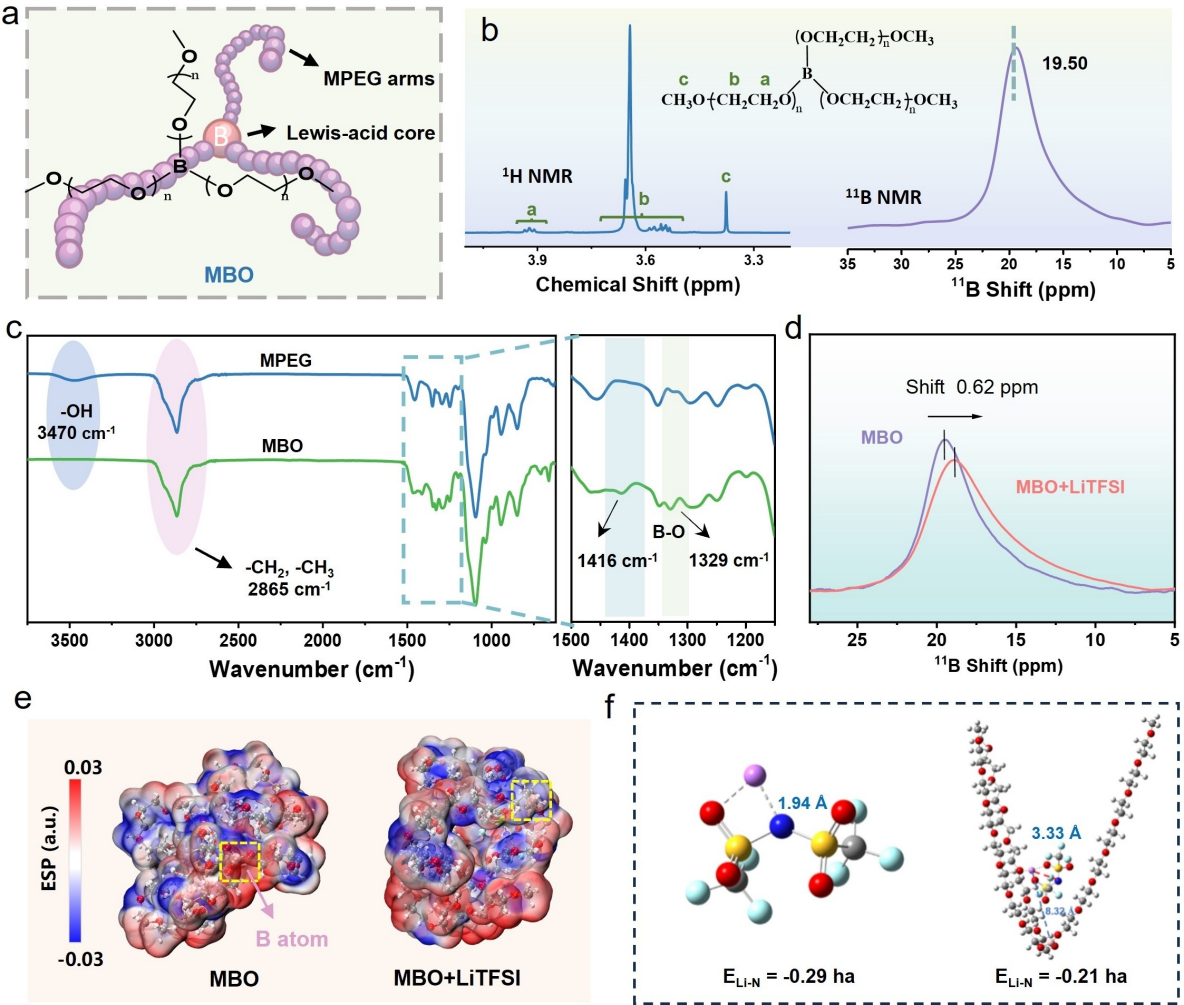
(a) The chemical structure of the designed MBO. (b) The ^1^H NMR and ^11^B NMR spectra of MBO. (c) FT‐IR spectra of MPEG and MBO. (d) ^11^B NMR spectra of MBO and MBO‐LiTFSI mixture. (e) The optimized geometry and electrostatic potential. (f) The calculated binding energy and bonding distance in a single LiTFSI (left) and MBO‐LiTFSI mixture (right).

Figure 2a illustrates the chemical structure of the MBO solid plasticizer, which consists of a Lewis‐acidic boron core and three linear MPEG oligomers arms. The corresponding synthesis described in Experimental section and Figure S2. The chemical structure of MBO was confirmed based on ^1^H nuclear magnetic resonance (NMR), ^11^B NMR, and Fourier transform infrared spectroscopy (FTIR) (Figure 2b, c and Figure S3). Signals in the range of 3.90–3.95 ppm in ^1^H NMR spectrum correspond to protons in the EO moiety near the borate (B−O‐CH_2_−), implying the successful substitution of the ‐OH groups in MPEG by the borates.^[27]^ A broad peak at 19.50 ppm in ^11^B NMR spectrum is consistent with the characteristic signal of tri‐oxygen coordinated boron.^[28]^ The FTIR spectrum of MBO further confirms the presence of boron and MPEG moieties. The peak at 665 cm^−1^ is assigned to the bending vibration of B−O, and the peaks at 1416 cm^−1^ and 1329 cm^−1^ belong to the stretching vibration of B−O.[^29^, ^30^] All these results demonstrated that the MBO has been successfully synthesized through a facile one‐step ester‐exchange reaction.

To understand the effect of MBO on LiTFSI due to Lewis acid‐base interaction between MBO and LiTFSI, ^11^B NMR was carried out on the corresponding mixture. Figure 2d shows that the chemical shift for boron decreased in ^11^B NMR spectrum after introducing LiTFSI, suggesting interaction formation between TFSI^−^ anions and B atoms in MBO.^[31]^ Figure 2e illustrates the optimized geometry and electrostatic potential (ESP) between MBO and LiTFSI calculated by density functional theory (DFT). They confirm that boron in MBO undergoes attractive interactions with the anion. As shown in Figure 2f, the introduction of MBO results in the reduced binding energy (−0.21 ha) and extended bonding distance (3.33 Å) of Li−N bond in LiTFSI, implying the weakening of Li‐TFSI interaction and an increasing tendency for Li^+^ dissociation.[^32^, ^33^] These experimental and computational results confirmed the presence of Lewis acid‐base interaction between the B atoms in MBO and TFSI^−^ anions, thereby contributing to higher ionic conductivity and Li^+^ transference number.

### The Physicochemical Properties of PCPE‐x Containing MBO

**Figure 3:**
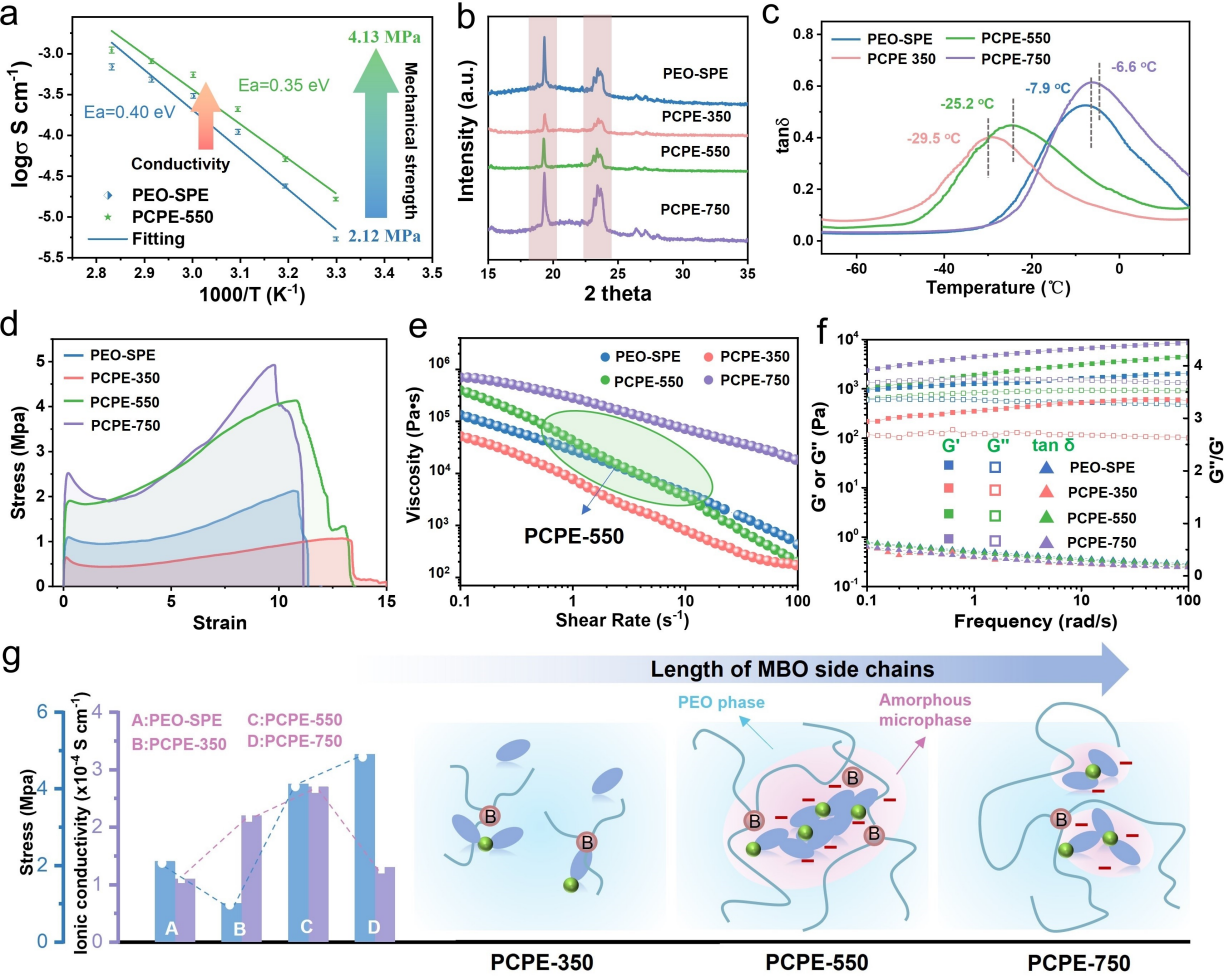
(a) Comparison of ionic conductivity of PEO‐SPE and PCPE‐550. (b) XRD, (c) DMA and (d) the stress‐strain curves of the PEO‐SPE, PCPE‐350, PCPE‐550, and PCPE‐750. (e) The relationship between apparent shear viscosity and shear rate of the polymer electrolytes. (f) Storage (G′)and loss (G“) moduli (Pa) and loss tangent (tan δ= G”/G′) versus the frequency of the polymer electrolytes. (g) Relationship between ionic conductivity and mechanical properties of PCPE‐x electrolytes using different MBO plasticizers that possess different length of MPEG side chains.

For PCPE preparation, desired amounts of MBO were dissolved in the PEO‐LiTFSI casting solution. The resulting PCPE membrane was obtained through vacuum drying, marked as PCPE‐x (where x represents the relative molecular weight of MPEG arms in the MBO). Scanning electronic microscope (SEM) images and corresponding energy dispersive spectroscopy analysis shown in Figure S4 and S5 suggested that the MBO plasticizer stays homogeneously distributed within the PEO‐LiTFSI matrix. Figure 3a depicts the comparison of ionic conductivity of original PEO‐based electrolyte (PEO‐SPE) and the PCPE‐550 based on electrochemical impedance spectroscopy (EIS) (Figure S6). As hoped for, the ionic conductivity of PCPE‐550 is increased compared with that of PEO‐SPE over the whole temperature range, with the conductivity of PCPE‐550 reaching 2.70×10^**−**4^ S cm^**−**1^ at 50 °C, showing a low activation energy of 0.35 eV. At the same time, the mechanical properties of PCPE‐550 were also enhanced compared to the PEO‐SPE. Both the mechanical strength and Young's modulus of PCPE‐550 increased, 4.13 vs. 2.12 MPa, and 1.84 vs. 1.12 MPa, respectively, according to stress‐strain curves and atomic force microscopy experiments (Figure S7 and 8). Such phenomenon is in stark contrast to other plasticizers (such as SN, ether‐based solvent, ionic liquid, etc.)‐containing SPEs.[^34^, ^35^, ^36^, ^37^] Plasticizers in general lower the glass transition point, the crystallization temperature, and the overall crystallinity, but at the cost of sacrificing mechanical properties. The increased mechanical properties of PEO after mixing with MBO are related to the supramolecular, salt mediated interactions of MBO and PEO, followed by a modified crystallization morphology, inducing reversible “physical crosslinks”, as discussed in the following part.

In order to optimize the structure of MBO and investigate its structure–property relationship on the ionic conductivity and mechanical properties of PCPE, MBO with different length of side chains were synthesized using the MPEG with molecular weight of 350 and 750. Their ^1^H NMR and ^11^B NMR analyses are provided in Figure S9 and 10. The X‐ray diffraction (XRD) analysis in Figure 3b, showed reduced intensity of characteristic peaks for PEO in PCPE‐350 and PCPE‐550, compared to that in PEO‐SPE, while the intensity increased for PEO in PCPE‐750. The reduced crystallinity of PCPE‐350 and PCPE‐550 also translates to both a significantly lower glass transition temperature (**−**51 °C) and melting points (44 and 46 °C, respectively) compared to native PEO and PCPE‐750 (T_g_= **−**44 °C and *T*_m_= 51 °C for both) as determined by differential scanning calorimeter (DSC) measurement and depicted in Figure S11 and Table S1. The trend of T_g_ values for different electrolytes obtained from dynamic thermo‐mechanical (DMA) analysis in Figure 3c is the same as that from DSC. We observe the plasticizing effect of MBO with its relatively short length of MPEG side chains. Already this polymer amorphization improves Li^+^ migration and ionic conductivity as measured for PCPE‐350 and PCPE‐550 (Figure S12).[^34^, ^38^] It is worth noting that compared with PCPE‐350 and PCPE‐550, PCPE‐750 showed an increased crystallinity and lower ionic conductivity, that is the effect if forming a more extended amorphous, percolating microphase is restricted to lower molecular weights of the tri‐arm star additive (which is due to mixing thermodynamics). The stress‐strain curves of SPEs in Figure 3d showed that the mechanical strength of PCPE‐350 is obviously reduced compared to the pure PEO‐SPE, while the experimental tensile strength for the samples PCPE‐550 and PCPE‐750 is higher than for the reference PEO‐SPE. The experimental results also disclose that the PCPE with MBO‐550 plasticizer hits the sweet spot where ionic conductivity and mechanical strength are simultaneously improved.

Based on the above analysis, we can speculate that it is microphase demixing between PEO crystalline phase and a Lewis acid‐base interaction‐based MBO‐salt containing amorphous microphase that allows simultaneous optimization of the two properties, as illustrated in Figure 3g. The crystalline, percolating subphase is responsible for the high mechanical strength, while the amorphous, also percolating domain with their improved mobility contributes to the higher ion mobility. In such percolating domain, anion aggregation was significant to result in formation of a unique ion transport channel composed of connected salt clusters.[^39^, ^40^] The formation of salt clusters restricted the movement of anions but effectively migrated Li^+^.^[41]^ The MBO‐550 with appropriate MPEG arm length, might have helped to assemble the chunky and segregated anion‐aggregate domains into connected salt clusters through Lewis acid‐base interactions, forming an amorphous MBO‐salt superstructure to facilitate Li^+^ transport. Additionally, the formed MBO‐salt assembly superstructure with a certain size can work as nanofillers, also further strengthening the PCPE‐550 electrolyte.

We therefore deal with a composite material with crystalline polymer walls and amorphous pores filled with conduction salt, while the MBO acts as a type of surfactant stabilizing a specific nanoscopic microphase morphology or “superstructure”. For instance, too much of MBO‐750 with its longer side chains takes part into crystallization and wall formation, leading to smaller and more separated amorphous salt cluster and the absence of plastination coupled to a decreased glass transition. This leads to a sharp decline of ionic conductivity.

To verify this microphase‐separated membrane formation hypothesis, rheological tests were conducted, and the results are shown in Figure 3e. PEO‐SPE with high molecular weight exhibit rheological properties of a non‐Newtonian viscous fluid shear‐yielding under external forces, with viscosity decreasing as shear rate increases.[^42^, ^43^] When the lower molecular weight MBO‐350 is added, the shear viscosity of the polymer decreases, and so does the elastic modulus. This is rather pure plastination. MBO‐750 with its longer length of MPEG side rather connects the primary PEO crystals and shows the highest plateau modulus and the highest Young's modulus, due to the as formed physical crosslinks. The rheological properties become most complex when the medium molecular weight MBO‐550 is added. The shear viscosity of PCPE‐550 is higher than PEO‐SPE at low shear rates, while is lower at high shear rates. This points to simultaneous local plastination while exposing a higher cross‐linking density visible at lower shear rates. Shear experiments allow to determine the storage modulus (G′) and loss modulus (G′′) of the PCPE‐550 and PCPE‐750. The values shown in Figure 3f are significantly increased, while the tangent value of loss angle (tan δ) stays less than 1, indicating a mainly solid‐like, elastic behavior.[^24^, ^44^] As the value of the plateau modulus linearly with the cross‐linking density, we can state that the number of cross‐links in PCPE‐550 is indeed twice as high as in the parental PEO‐SPE, in spite of adding a plasticizer. Next, we further investigated the ionic conductivity and mechanical properties of PCPE with different MBO‐550 contents, the results and the detailed discussion are shown in Figure S13 and S14. Only 5 wt % of the MBO‐550 modifier obviously promotes salt bridged, microphase‐demixed morphology, and a significantly improved and hardened supramolecularly extended polymer network is formed, thus achieving considerable both ionic conductivity and mechanical properties. Moreover, the PCPE‐550 with appropriate MBO content (5 wt %) also displays good thermal stability (Figure S15) and excellent mechanical flexibility (Figure S16), which makes it practically appealing.

### Exploring the Transport of Li^+^ in the PCPE‐550

**Figure 4:**
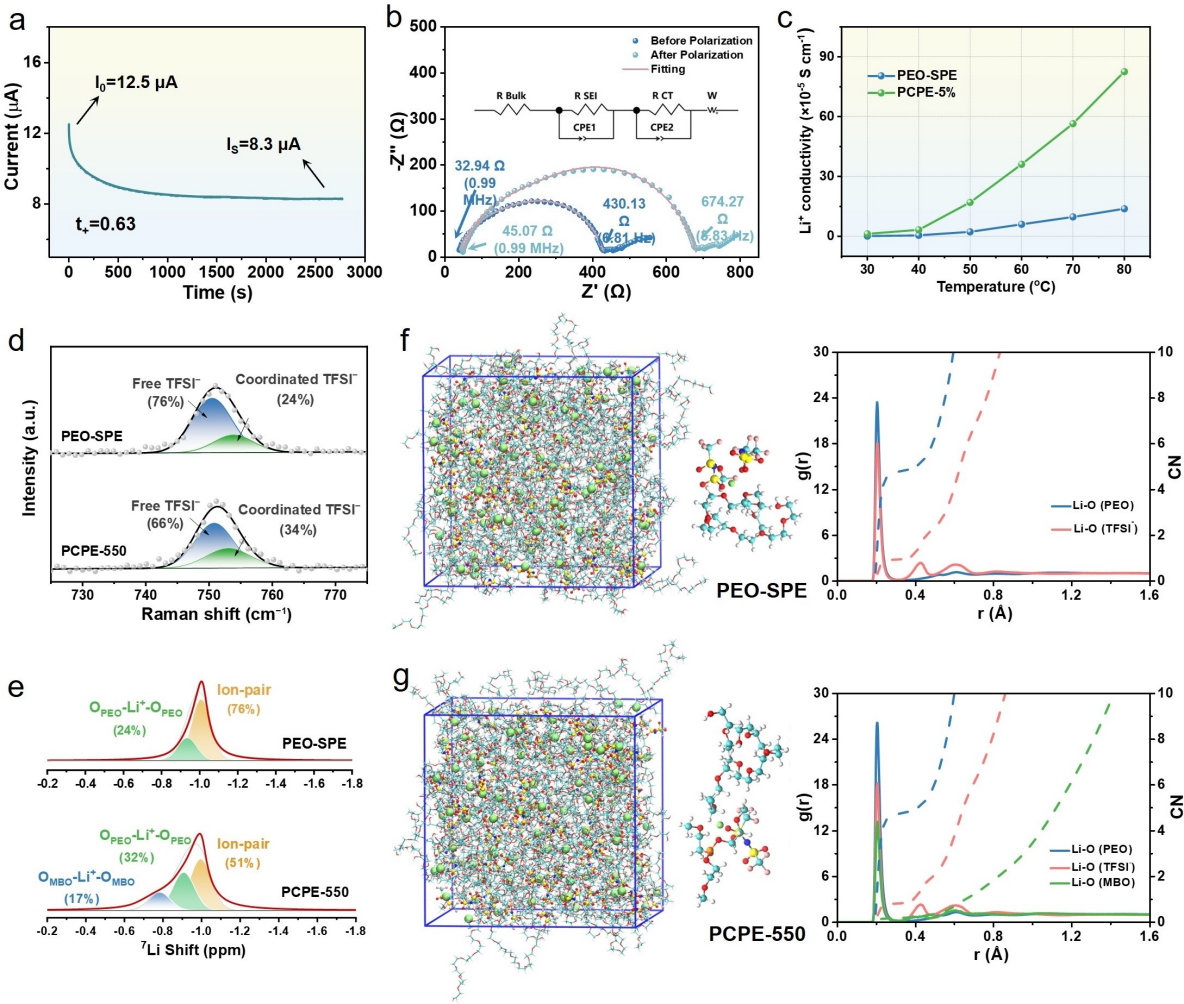
(a) Polarization curve and (b) fitted EIS before and after polarization of PCPE‐550. (c) Li^+^ conductivity of PEO‐SPE and PCPE‐5 wt %. (d) Raman spectra and (e) ^7^Li solid‐state NMR spectra of the PEO‐SPE and PCPE‐550. The Li−O radial distribution functions (RDF) of (f) PEO‐SPE and (g) PCPE‐550.

To shed more light on the specific role of MBO and explore the Li^+^ transport behavior PCPE, we investigated charge transfer properties in PCPE‐550 and compared to that of PEO‐SPE. The lithium‐ion transport number (t_+_) was evaluated through chronoamperometry combined with EIS; the fitting and the calculation details of the impedance spectra are included in Table S2~S5, respectively. The obtained t_+_ based on this method is 3 times higher for PCPE‐550 (Figure 4a and b) than for the reference PEO‐SPE (Figure S17), indicated that PCPE‐550 has better lithium‐ion transport than PEO‐SPE. To be clear, since the PEO‐SPE and PCPE‐550 are concentrated systems containing ion complexes, the t_+_ values obtained here are actually ‘‘cationic fractions’’ of the current, rather than Li^+^ transference number.^[45]^ Benefiting from the high ionic conductivity (Figure 3a) and t_+_, the PCPE‐550 possesses a higher lithium transport capability than PEO‐SPE (Figure 4c). Besides increased ion mobility, we also attribute this to the change of ion structure promoted by MBO‐550 as some type of microphase stabilizer, forming and improving interconnected ion transport paths. Figure S18 shows that indeed the electrochemical stability window has been widened up to 5.08 V (vs. Li^+^/Li), indicating a superior oxidation resistance under high‐voltage state in comparison to native PEO (4.12 V) (vs. Li^+^/Li). This is only possible when the current is transported in a MBO‐salt assembly superstructure of salt‐clusters in PEO. In addition, the Lewis acid‐base interaction between boron sites in MBO and oxygen atoms in PEO chains may also be responsible for the improved electrochemical stability.

To obtain a better insight into the interaction between TFSI^−^ anions and MBO as well as the overall ion coordination environment, Raman and solid‐state ^7^Li NMR were performed. The results extend the simplified picture that the interaction between TFSI^−^ and B atoms only promotes dissociation of Li salts. As shown in Figure 4d, the proportion of free TFSI^−^ in PCPE‐550 (66 %) is lower than that of PEO‐SPE (76 %), and the peak of coordinated TFSI^−^ in the PCPE‐550 was left shifted relative to PEO‐SPE. This suggested that the MBO effectively captures free TFSI^−^ and negatively charged anion complexes through Lewis acid‐base interaction, have helped to assemble the chunky and segregated anion‐aggregate domains into connected salt clusters, thereby forming a MBO‐salts superstructure to facilitate Li^+^ transport.^[46]^ As membrane composition gives about 35 TFSI^−^ or Li ions per boron moiety, the overall story however must be more complicated. ^7^Li solid‐state NMR spectra, depicted in Figure 4e, revealed three peaks, corresponding to three different Li^+^ environments. One at −1.02 ppm is usually attributed to the dissolved ion pair, while the second at −0.96 ppm is related to Li^+^ coordinated with oxygen in PEO. After addition of MBO, there is a slight increase of the content of Li^+^ coordinated with oxygen in the PCPE‐550 (32 %) compared to the PEO‐SPE (24 %), in accordance with Raman results. Moreover, a third peak appears at −0.75 ppm, which could be attributed to the coordination of EO group in near the center of MBO with Li^+^, and this conclusion was further supported by the extra ^7^Li NMR spectrum of MBO environment with only LiTFSI salt (Figure S19). MBO thereby does not act only on the molecular level, but creates a MBO‐salt assembly superstructure where about 49 % of all lithium ions are NMR‐downshifted and more mobile. The enhanced mobility of Li^+^ is therein explained by an increased amount of interconnected ion transport paths located off the PEO chains and near the MBO and salt clusters.[^47^, ^48^, ^49^] We employed molecular dynamics (MD) simulations to reveal further details on the change of Li^+^ transport in the PCPE‐550 due to the modification of its interactions with the diverse oxygen atoms. As expected, the Li−O radial distribution functions (RDF) obtained from MD simulations show that there is a lower average number of ether oxygen in the first solvation shell of Li^+^ in PCPE‐550 than in PEO‐SEP (4.70 vs. 4.76). This implies that Li^+^ in the PEO‐SPE complex is more strongly solvated (Figure 4f and 4 g, Table S6). A new, relatively weak peak of MBO occurs at 0.2 Å (the first solvation shell), indicated that the MPEG arms of MBO participate in lithium‐ion conduction through coordination interaction with Li^+^, the new migration path. Thereby, also in modelling, the weakened solvation shell around Li^+^ after the addition of MBO in the PCPE‐550 complex sets a base for its enhanced ion transport kinetics.[^50^, ^51^, ^52^]

### The Li/PCPE‐550 Interfacial Stability in Symmetric Li/Li Cells

**Figure 5:**
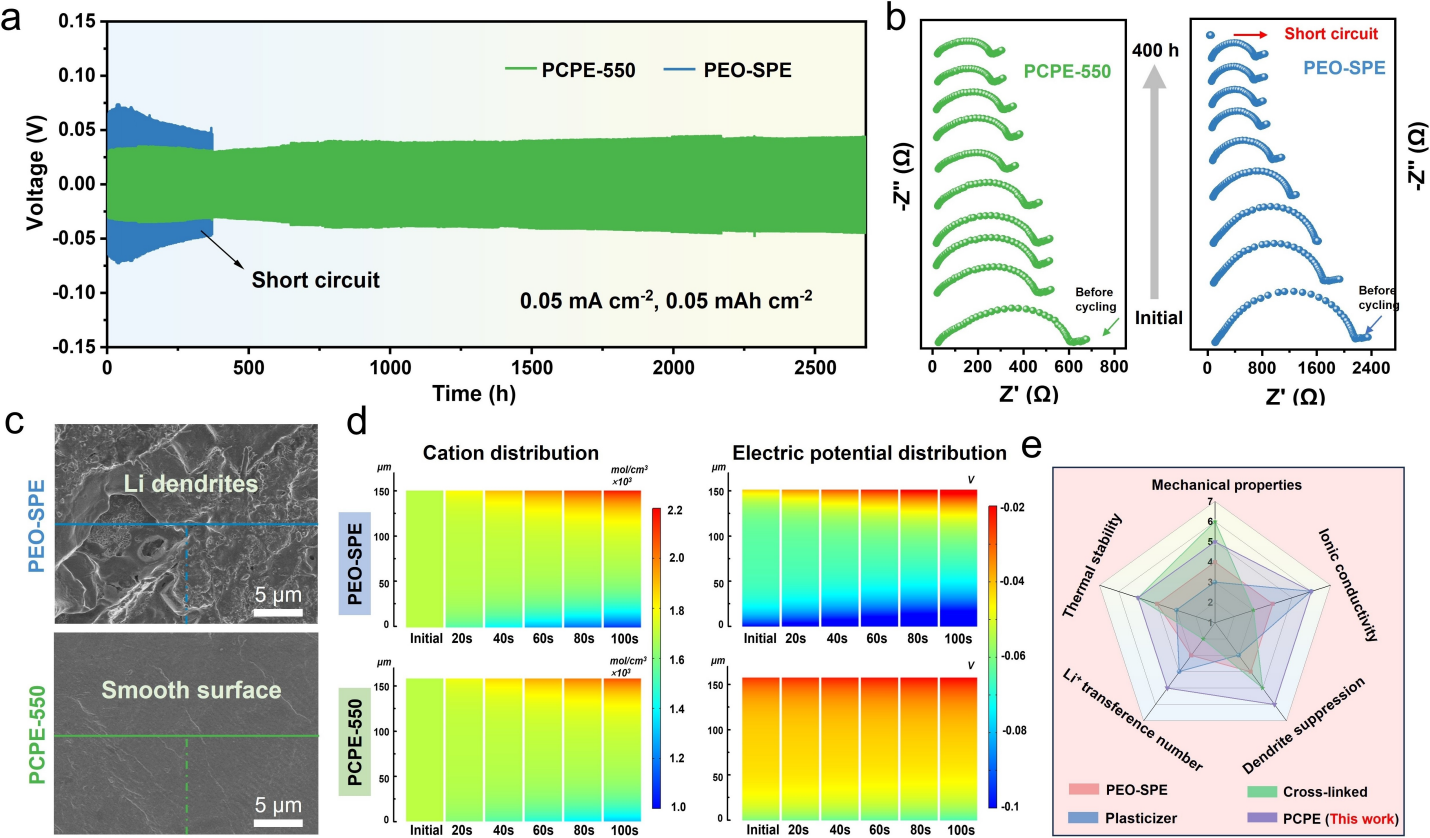
(a) Long‐term cycling performance of Li/Li symmetric cells using PCPE‐550 and PEO‐SPE at 0.05 mA cm^−2^ and 0.05 mAh cm^−2^. (b) EIS evolution of Li/Li symmetric cells with the PCPE‐550 and PEO‐SPE within initial 200 cycles. (c) SEM images of the cycled Li anode surfaces disassembled from symmetric cell. (d) The Li^+^ concentration distribution and electric field distribution in the PCPE‐550 and PEO‐SPE electrolytes during the Li plating process. (e) Radar plots of the performance comparison of different SPEs.

To check for potential improvements in the nanocomposite electrolyte layer performance, symmetric Li/Li cells were assembled to evaluate the interface film stability of the PCPE‐550 against Li metal anode. Figure 5a shows the long‐term cycling performance of Li/Li symmetric cells using PCPE‐550 and PEO‐SPE at 0.05 mA cm^−2^ and 0.05 mAh cm^−2^. It is observed that the symmetric cell using the pure PEO‐SPE material (as a reference of the state of the art) has a relatively high initial overpotential with obvious voltage fluctuation, and a short circuit occurred after less than 400 h. By contrast, Li/PCPE‐550/Li symmetric cell shows excellent cycling stability for over 2600 h without short‐circuiting. We relate this excellent mechanical strength and very good interfacial compatibility with the Li metal, effectively suppressing dendrite formation. Even at high current density, the Li/PCPE‐550/Li cell still exhibits steady polarization with low overpotentials (Figure S20 and 21), a direct consequence of the enhanced mobility kinetic of Li^+^. The superior stability of the polymer electrolyte PCPE‐550 film on Li metal is further investigated by time‐resolved impedance spectroscopy. As seen from Figure 5b, the overall cell resistance of the Li/Li symmetric cell with PEO‐SPE electrolyte continuously shifts to lower values followed by a dramatic decrease of overall resistance, indicative of cell failure due to short circuits, presumably by dendrites. In sharp contrast, the symmetric cell assembled with PCPE‐550 electrolyte demonstrates more constant but relatively low resistance during long‐term cycling. Due to the superiority of PCPE‐550 in enhancing the stability toward Li metal, a better rate capability was achieved for Li/PCPE‐550/Li symmetric cell in the range of current densities between 0.05 to 1 mA cm^−2^ (Figure S22).

After extensive cycling tests under a current density of 0.05 mA cm^−2^, compared with initial Li foil, we find as expected by SEM porous lithium deposits and serious Li dendrites on the reference PEO‐SPE based Li metal surface (Figure S23 and Figure 5c). On the contrary, the Li metal cycled with the PCPE‐550 presented in SEM a much flatter morphology under the same conditions, indicating stable and reversible plating/stripping process and excellent mechanical resilience at interface (Figure S24). The change in surface morphology and roughness of cycled Li anodes analyzed by a 3D confocal laser scanning microscope (CLSM) in Figure S25 further confirms the enhanced interfacial stability of PCPE‐550.^[46]^ In order to quantitatively evaluate the contribution of high Li^+^ transport number of PCPE‐550 to interfacial stability resulting from the decreased concentration gradient and electric field gradient, modelling with finite‐element method (FEM) using COMSOL Multiphysics software was applied. The ion concentration distribution (including Li^+^ ions and TFSI^−^ anions) and electric field distribution at different cycling stages are depicted in Figure 5d and Figure S26. PCPE‐550 presents a relatively uniform ion concentration distribution with small concentration gradients of Li^+^ during the cycling process, in stark contrast to these of PEO‐SPE. The diverse properties of PCPE‐550, the conventional reference PEO‐SPE, and currently reported advanced SPE systems, are visually displayed in a radar plot in Figure 5e, showing all‐around improvements of PCPE‐550 when adding the functional MBO, acting both as a plasticizer and a kind of surfactant stabilizing a bi‐continuous nanocomposite structure with crystalline PEO walls and liquid, ion containing transport pores (Table S7). As that, PCPE‐550 effectively overcomes the discrepancy between enhancing mechanical robustness and improving ionic conductivity in SPEs. We consider this as a major step forward for the design of advanced practically SPE systems.

### Electrochemical PROPERTIEs of PCPE‐550 in a Model All‐Solid‐State Battery

**Figure 6:**
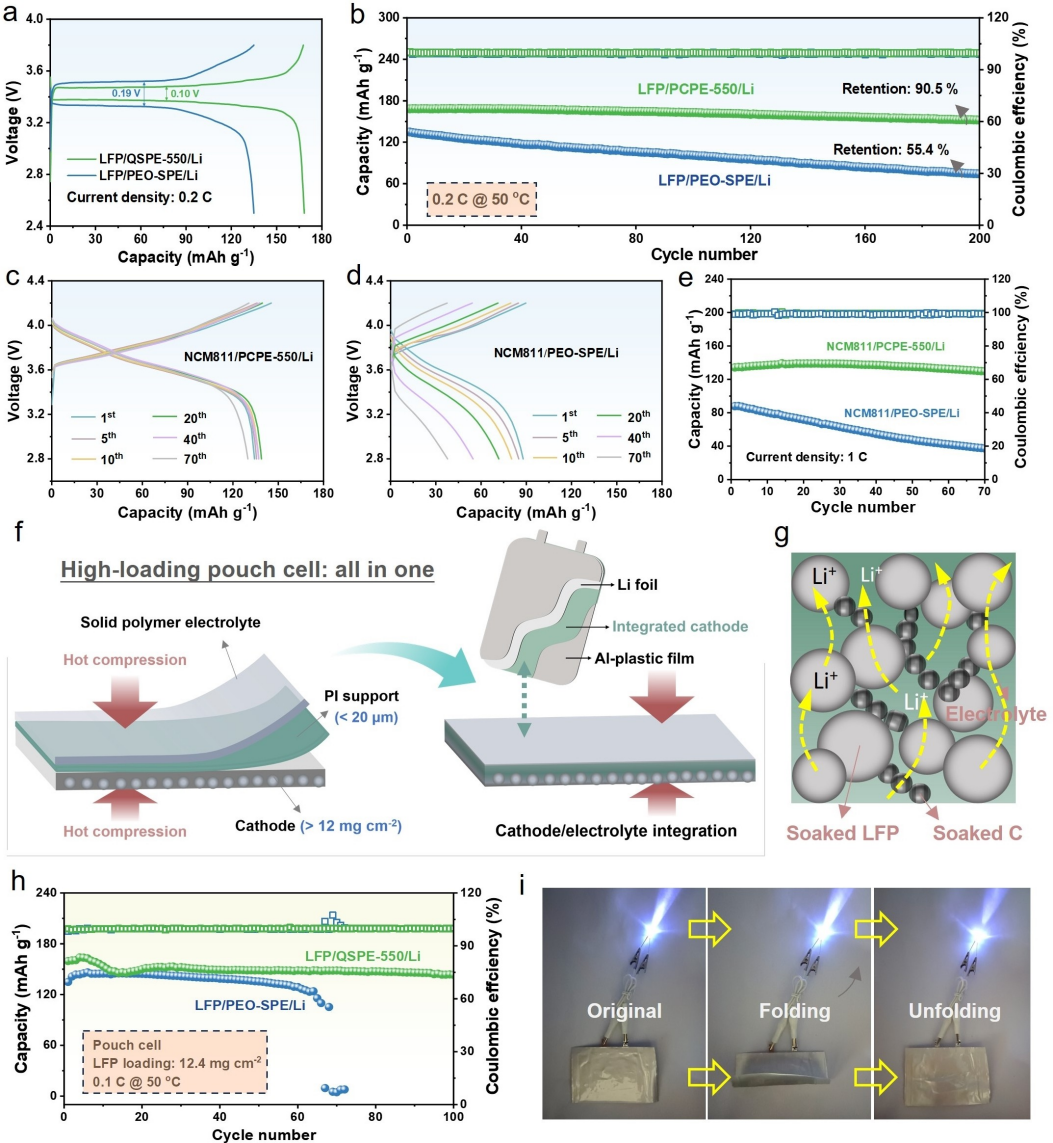
(a) Charge/discharge profiles of the first cycle and (b) long‐term cycling performance of all‐solid‐state LFP/PCPE‐550/Li and LFP/PEO‐SPE/Li cells under 0.2 C at 50 °C. Charge/discharge profiles of all‐solid‐state NCM811/Li cells using (c) PCPE‐550 and (d) PEO‐SPE electrolytes at 1 C. (e) Cycling performance of all‐solid‐state NCM811/Li cells under 1 C. (f) Schematic diagram of the preparation route of integrated LFP cathode and all‐in‐one LFP/Li pouch cell. (g) Internal structure of the integrated cathode with high LFP mass loading. (h) Cycling performance of all‐solid‐state LFP/Li pouch cells under 0.1 C at 50 °C. (i) Digital photos of LFP/PCPE‐550/Li pouch cell lighting light‐emitting diode.

ASSLMBs using PCPE‐550 and PEO‐SPE were constructed by coupling it to a Li metal anode and a LiFePO_4_ (LFP) cathode. As shown in Figure 6a, compared with LFP/PEO‐SPE/Li cell, the LFP/PCPE‐550/Li delivers a much higher discharge capacity of 167.2 mAh g^−1^ with a smaller voltage polarization at 0.2 C, 50 °C, indicating rapid Li^+^ migration and low interface resistance. Clearly, from Figure 6b and Figure S27, the all‐solid‐state LFP/PCPE‐550/Li cell displays excellent long‐term cycling stability, possessing a capacity retention up to 90.5 % after 200 cycles, while the PEO‐SPE based cell shows rapid capacity decay with cycling, and only with 55.4 % capacity retention. Excitingly, even at 30 °C, the all‐solid‐state LFP/PCPE‐550/Li full cell still demonstrates considerable electrochemical properties, delivering here an initial capacity of 136.5 mAh g^−1^ and a high‐capacity retention of 88.9 % after 200 cycles at 0.1 C (Figure S28 and 29). This is superior to most of currently reported works (Table S8). Additionally, due to the positive effect of PCPE‐550 on cell performances, the PCPE‐550 based cell also demonstrated considerable rate capability (Figure S30 and 31). The enhanced cycling stability and rate capability is attributed to the high Li^+^ conductivity and mechanical properties of PCPE‐550, which support rapid Li^+^ migration kinetics and a favorable electrode/electrolyte interface. Next, to demonstrate the advantages of PCPE‐550 for practical application, the all‐solid‐state batteries coupled with high‐voltage LiNi_0.8_Co_0.1_Mn_0.1_O_2_ (NCM811) cathode were assembled and tested in the voltage range of 2.8–4.2 V at °C. Figure S32 and S33 presents the cycling performance and charge/discharge profiles of the assembled NCM811/PCPE‐550/Li and NCM811/PEO‐SPE/Li batteries at 0.5 C. The cell using PCPE‐550 electrolyte delivers a higher discharge capacity of 145.2 mAh g^−1^with relatively small polarization, which outclasses those of the PEO‐SPE based batteries. Even under a high current density of 1 C, the NCM811/PCPE‐550/ Li battery exhibits a capacity retention of 96.7 % after 70 cycles (Figure 6c‐e). However, the NCM811/PEO‐SPE/Li displays a rapid capacity degradation. Above results demonstrates the great potential of PCPE‐550 electrolyte in achieving high energy‐density Li metal batteries.

To further demonstrate the feasibility of PCPE‐550 in practical applications, a LFP /Li pouch cell with high cathode mass loading (>12 mg cm^−2^) was constructed and tested at 50 °C. In this section, in order to solve the problem of sluggish Li^+^ migration kinetics inside thick cathode caused by the poor contact between active material and solid electrolyte, the SPEs were melted and infiltrated into porous polyimide (PI) substrate membrane that was placed on the cathode surface to form an integrated cathode by a solvent‐free hot‐pressing technique (Figure 6f). As illustrated in Figure 6g, this process can reduce the interface contact impedance of cathode and electrolyte, forming continuous and rapid Li^+^ transport paths inside the high‐loading cathode. It helps to decrease the thickness of SPEs, which is meaningful to implement high energy and power density of full cells. From Figure S34 and 35, the integrated cathode displays an all‐in‐one structure with high flexibility after hot‐pressing treatment, and the PCPE‐550 was fully permeated into porous PI membrane, and a LFP cathode with continuous polymer phase was formed between electrode and electrolyte. The lower charge‐transfer resistance of the LFP/PCPE‐550/Li cells assembled with the integrated cathode was compared to the non‐integrated cathode in Figure S36 and further confirmed the favorable cathode/electrode interfaces in the integrated cathode. Figure 6h shows the cycling properties of pouch cells using high‐loading integrated cathode and different electrolytes. It can be seen that the cell using PCPE‐550 displayed much better cycling stability with an average discharge capacity of 152.4 mAh g^−1^ at 0.1 C within the first 100 cycles, which is far superior to other reported ASSLMBs (Table S9). Moreover, the pouch cell still continuously lights up light‐emitting diodes under folding and spreading conditions (Figure 6i). These mechanical abuse tests suggest that the PCPE‐550 composite membrane promotes not only excellent cycling performance, but also high mechanical resilience and thereby safety at the practical pouch cell level. In a word, the PCPE‐550 with its unique supramolecular mesostructure and intermolecular interactions enables rapid Li^+^ migration kinetics, low interfacial resistance, and enhanced mechanical performance, thus contributing to a significantly promoted competitive performance of an all‐solid‐state battery based on this material.

## Conclusion

In summary, we manufactured a supramolecularly extended/cross‐linked polymer electrolyte (PCPE) where a boron containing star‐shaped plasticizer (MBO) added in about 5 wt % stabilizes a favorable microphase separation into a semicrystalline PEO matrix phase and liquefied, salt‐cluster‐containing nanochannels. We show that in this PCPE, the MBO with abundant Lewis‐acid sites reduces the liquid subphase PEO glass transition while inducing the formation of MBO‐salt assembly superstructure with interconnected nanochannels for rapid Li^+^ percolation. The combination results in significantly improved Li^+^ conductivity. An optimized length of the tri‐arm star MBO allows also to control the crystallization of the PEO phase, which at intermittent MBO size gives a more elastic material with higher mechanical roughness at the same time, which we attribute to both salt‐induced and topological cross‐linking of the primary PEO nanocrystals. The resulting partially crystalline nanocomposite thereby can realize higher hardness, flexibility, and high Li^+^ mobility at the same time. With this PCPE, a Li metal anode was achieved with an outstanding long‐term cycling stability, being dendrite‐free over 2600 h while enabling high‐rate and long‐term cycling at room temperature. Additionally, an ASSLMB pouch cell with a well‐designed, integrated, high‐loading LFP cathode was realized which showed outstanding electrochemical performance. We believe that the present work introduces an effective strategy to overcome the dilemma between mechanical robustness and ionic conductivity in SPEs by controlling the nano‐ and microphases, opening the door of practical application of SPEs in ASSLMBs for next‐generation energy storage technologies.

## Conflict of Interests

The authors declare no conflict of interest.

1

## Supporting information

As a service to our authors and readers, this journal provides supporting information supplied by the authors. Such materials are peer reviewed and may be re‐organized for online delivery, but are not copy‐edited or typeset. Technical support issues arising from supporting information (other than missing files) should be addressed to the authors.

Supporting Information

## Data Availability

The data that support the findings of this study are available on request from the corresponding author. The data are not publicly available due to privacy or ethical restrictions.
