# Supplementary material for: Mixing Functionality in Polymer Electrolytes: A New Horizon for Achieving High‐Performance All‐Solid‐State Lithium Metal Batteries
Source: Angew Chem Int Ed Engl. 2025 Feb 28;64(18):e202422169. doi: 10.1002/anie.202422169 (PMC12036809; doi:10.1002/anie.202422169)
Supplement: Supplementary file 1 — Supporting Information [file ANIE-64-e202422169-s001.pdf]

Angewandte Chemie
Eine Zeitschrift der Gesellschaft Deutscher Chemiker

Supporting Information

Mixing Functionality in Polymer Electrolytes: A New Horizon for Achieving High-Performance All-Solid-State Lithium Metal Batteries

Y. Ren, S. Chen, M. Odziomek, J. Guo, P. Xu, H. Xie, Z. Tian*, M. Antonietti*, T. Liu**

Supporting Information

Mixing Functionality in Polymer Electrolytes: A New Horizon for Achieving High-Performance All-Solid-State Lithium Metal Batteries

Yufeng Ren, Suli Chen, Mateusz Odziomek, Junhong Guo, Pengwu Xu, Haijiao Xie, Zhihong Tian,* Markus Antonietti,* Tianxi Liu**

Y. F. Ren, Dr. S. L. Chen, J. H. Guo, Dr. P. W. Xu, Prof. T. X. Liu

The Key Laboratory of Synthetic and Biological Colloids, Ministry of Education, School of Chemical and Material Engineering, Jiangnan University, Wuxi, 214122, P. R. China

E-mail: chensl@jiangnan.edu.cn; txliu@jiangnan.edu.cn

H. J. Xie

Hangzhou Yanqu Information Technology Co., Ltd., Hangzhou, 310003, P. R. China

Prof. Z. Tian

Engineering Research Center for Nanomaterials, Henan University, Kaifeng 475004, P. R. China

E-mail: zhihong.tian@henu.edu.cn

Dr. M. Odziomek, Prof. M. Antonietti

Department of Colloid Chemistry, Max Planck Institute of Colloids and Interfaces, Am Mühlenberg 1, Potsdam 14476, Germany

E-mail: markus.antonietti@mpikg.mpg.de

Experimental Section

Materials

Borane tetrahydrofuran complex solution (BH_3/THF, 1 M in THF), methoxypolyethylene glycol (MPEG, Mn=350, 550, 750), polyethylene oxide (PEO, Mw=1×10^6), and anhydrous acetonitrile (ACN, 99.8%) were purchased from Aladdin Chemical Reagent Co. Ltd., China. Bis(trifluoromethane)sulfonimide lithium salt (LiTFSI), lithium iron phosphate (LFP), $LiNi_{0.8}Co_{0.1}Mn_{0.1}O_2$ (NCM811)，N-methyl pyrrolidone (NMP) were purchased from Guangdong Canrd New Energy Technology Co. Ltd., China. Polyvinylidene fluoride (PVDF) and Super P were achieved from Sigma-Aldrich. All the chemicals were used as received without further purification.

Synthesis of multi-arm boron-contained oligomer (MBO)

The MBO was synthesized by the reaction of MPEG and borane (BH_3). First of all, 18 mmol of MPEG was dissolved in 20 mL of ACN under nitrogen atmosphere and then the mixtures were stirred at 45 ^oC for half an hour. After that, 12 mmol of BH_3/THF (containing 12 mmol Borane, using excess BH_3 assures all the raw MPEG can be reacted) was added at the rate of drop per five seconds by a constant pressure dropping funnel. The reaction was held under reflux for 36 h. After the reaction procedure, the ACN and BH_3 were removed by a rotary evaporator at 70 ^oC. Since the MBO is sensitive to water, the product was then transferred into an Ar-filled glovebox and stored.

Preparation of PCPE and PEO-SPE Membrane.

The PEO and the stoichiometric LiTFSI salt were dissolved in ACN to form a uniform solution, with a molar ratio of ethylene oxide unit and lithium of 12:1. Then, different ratios (2 wt%, 5 wt%, 8 wt%, 10 wt% of PEO) of the obtained MBO were added into the above solution. The mixture was stirred for 24 h to form a homogeneous colloid. After that, cast the above mixture onto a Teflon substrate and heat it at 60 ^oC for 12 h to let the solvent evaporate and form an electrolyte membrane. Herein the PEO/MBO/LiTFSI (PCPE) membrane was obtained. For comparison, the MBO-free

(PEO/LiTFSI, PEO-SPE) membrane was prepared, following the above steps without adding MBO. All preparation processes of the solid polymer electrolytes (SPEs) were operated in an Ar-filled glove box (H_2O < 0.01 ppm, O_2 < 0.01 ppm).

Preparation of Cathodes and Assemble of Batteries

The coin cells were assembled with LFP and NCM811 cathodes, polymer electrolyte membranes and lithium metal inside CR-2032 type case in an argon-filled glovebox (H_2O <0.01 ppm, O_2 <0.01 ppm). The LFP cathode was prepared using a slurry composed of 80 wt.% LFP active material, 10 wt.% Super P, and 10 wt.% PVDF, with NMP solvent used to ensure uniform distribution of the components. Subsequently, the slurry was cast onto an aluminum current collector using a doctor blade technique to achieve a uniform coating. The coated current collector was dried under vacuum at 80 ^{o}C for 12 h to remove the solvent and any residual moisture. Then, it was cut into 12 mm pieces for battery assembly. And the NCM811 cathode was prepared by a similar process. The mass loading of LFP active material for coin cells was 1.0-1.2 mg cm^{-2}, and 12.4 mg cm^{-2} for pouch cells. Similarly, the same weight ratio was applied to fabricated composite NCM811 cathode with a mass loading of about 1-1.2 mg cm^{-2}.

For pouch cell assembly, before cell assembly, an integrated cathode was prepared by a hot-pressing method in an Ar-filled glove box. In detail, a thin and porous polyimide (PI, thickness <30 μm) substrate membrane is pre-bonded to the cathode surface to avoid cell short-circuit. And then, a prepared electrolyte membrane was placed upon PI matrix, under the conditions of 100 ^{o}C and 16 MPa pressure, the electrolyte melts are fully permeated into porous PI matrix and cathode by controlling hot-pressing time, an integrated cathode with dense structure is successfully fabricated

through the hot-pressing process.

Material characterization

^{1}H and ^{11}B-nuclear magnetic resonance (Bruker AVANCE III HD 400 MHz) were used to evaluate the chemical structure. Fourier transform infrared spectrometer (FTIR, Nicolet 6700) was conducted to characterize chemical structure of samples. Scanning electron microscopy (SEM, Hitachi S-4800 s) with an energy dispersive spectrometer (EDS) was used to confirm the morphologies of samples. Atomic force microscopy (AFM, Bruker Dimension Icon, Germany) images were recorded using a tapping mode. X-ray diffraction (XRD, Bruker D8) with a Cu Kα X-ray source (λ = 1.542 Å) was recorded in the range of 10° to 80°. The solid-state ^{7}Li magic-angle-spinning (MAS) NMR experiments were performed on a Bruker Avance Neo 400WB system with 3.2 mm DVT probe. Differential scanning calorimeter (DSC, DSC3), thermal gravimetric analysis (TGA, TGA/DSC1/1100SF), and dynamic thermomechanical analysis (DMA, Q800) were used to evaluate the thermal behavior of the samples. The tensile strength of electrolytes is further measured on a universal tensile testing machine. The rheological measurements were performed using oscillatory shear rheology (MCR302e rheometer) with parallel-plate geometry (diameter of 2.5 mm). The oscillatory shear experiments were carried out at a strain of $\gamma = 10\%$ and a shear rate of $\omega = 0.1\sim100$ rad/s was used at 50 °C. Shear-thinning tests were carried out with a progressively increasing shear rate from 1×10^{-1} to 1×10^{2} s^{-1}.

CLSM is employed to assess the surface morphology of samples, utilizing a 658 nm laser as the detection light source. This laser is focused onto the sample surface via an optical system and an objective lens. An X-Y scanning optical system is then utilized to scan the image area within the field of view. The laser light reflected from the sample surface is detected by a CCD camera, which collects the laser intensity data. Subsequently, the objective lens is driven by a Z-axis actuator to perform repeated scans of the image, thereby obtaining the laser intensity for each pixel at every position along the Z-axis. The collected laser intensity data is subsequently reconstructed using 3D imaging software (VK-H1XAC, Keyence, Japan), yielding the surface three-

dimensional image and height distribution of the sample. Prior to conducting surface analysis, the electrolyte that adheres to the surface of the lithium metal is carefully removed in an Ar-filled glove box. Subsequently, a small, intact section of the lithium metal surface, measuring 250 × 204 μm, is extracted for CLSM. The peeling procedure has negligible impact on the sample surface.

The crystalline calculation equation is as follows:

$$X_c = \frac{\triangle H_f}{\triangle H} \times \frac{1}{w_f} \times 100\% \qquad (1)$$

Where X_c is the crystallinity of SPE membrane, $\triangle H_f$ is the integral value of melting enthalpy, $\triangle H$ is the melting enthalpy of 100% crystallized PEO which value at 213.7 J/g, w_f is the weight ratio of PEO.[1]

Electrochemical measurements

Ionic conductivities of the SPEs were tested by AC impedance spectroscopy using a CHIE660E electrochemical workstation with temperatures ranging from 20 to 80 ^{o}C. The SPEs were sandwiched between two stainless-steel electrodes and the EIS was obtained over a frequency range from 0.01 Hz to 10^6 Hz with a potential amplitude of 10 mV. The ionic conductivity (σ, S cm^{-1}) was calculated according to the equation:

$$\sigma = L/_{RS} \qquad (2)$$

where L (cm) and S (cm^2) stand for the thickness of SPEs and the area of SS electrode, respectively. R (Ω) refers to the bulk resistance of the electrolyte membrane measured by EIS.

Transport number (t_+) measurements of the SPEs was measured by the combination of EIS analysis and DC polarization with Li/Li symmetric cells, and the Bruce-Vincent equation for calculation is as follows:

$$t_+ = \frac{I_s\,(\Delta V - I_o R_0)}{I_0\,(\Delta V - I_s R_s)} \qquad (3)$$

where the applied potential ΔV is 10 mV, I_0 and I_s refer to the initial and stable current, R_0 and R_s represent interfacial resistance before and after polarization, respectively. The electrochemical stability of the SPEs was determined by linear sweep voltammetry (LSV) using Li/SPEs/SS cells and was carried out on a CHIE660E electrochemical

workstation at a scanning rate of 10 μV/s.

The cycling stability of Li/SPEs/Li and ASSLMBs were conducted using the multichannel battery testing systems (Land CT2001A). The cycle performance of LFP/SPEs/Li cells were tested between 2.5 and 3.8 V, while NCM811/SPEs/Li cells were tested at between 2.8 and 4.2 V. The C rates in all of the electrochemical measurements are defined based on 1 C=170 mA g^{-1} (LFP) and 1 C=180 mA g^{-1} (NCM811).

Computational details

Density functional theory (DFT) calculations were performed using Gaussian 16 package, B3LYP[2] density functional and 6-31+g(d) basis set were used for all calculations, the DFT-D3 method was used to describe dispersion correction in all calculations. The binding energy (E_b) was calculated by the following equation[3]:

$$E_b = E_{PolyEO-LiTFSI} - E_{PolyEO} - E_{Li^+} - E_{TFSI^-} \qquad (4)$$

Where $E_{PolyEO-LiTFSI}$ is the total energy of the structural PolyEO-LiTFSI, E_{PolyEO} is the energy of a single PolyEO molecule, E_{Li^+} is the energy of a single lithium ion, and E_{TFSI^-} is the energy of a single $TFSI^-$ ion, respectively.

The conformation search of molecule which contain more than 250 atoms was performed using xtb[4] and molclus software. The geometries were fully optimized at GFN2-xTB level[5] without any structural constraints. The electrostatic potential (ESP) figures were calculated using the Multiwfn[6] 3.8 program visualized by visual molecular dynamics (VMD)[7] software.

Simulation of ion concentration and electric potential distribution: The concentration distributions of Li^+ and $TFSI^-$ ions, as well as the potential distribution of the electrolyte, were investigated via finite element method (FEM) using COMSOL Multiphysics. The quasi-single ion conduction model was conducted FEM simulation. Charge transport in electrodes and electrolytes was analyzed by Ohm's law. Ion transport in spherical particle electrodes was evaluated across Fick's Law. And the plating/stripping of Li^+ was described by Butler-Volmer dynamical equation. All the parameters related to the electrolyte were the practical measured values. The applied

current density was 0.056 mA cm^{-2}.

Figure S1~S36, Table S1~S9

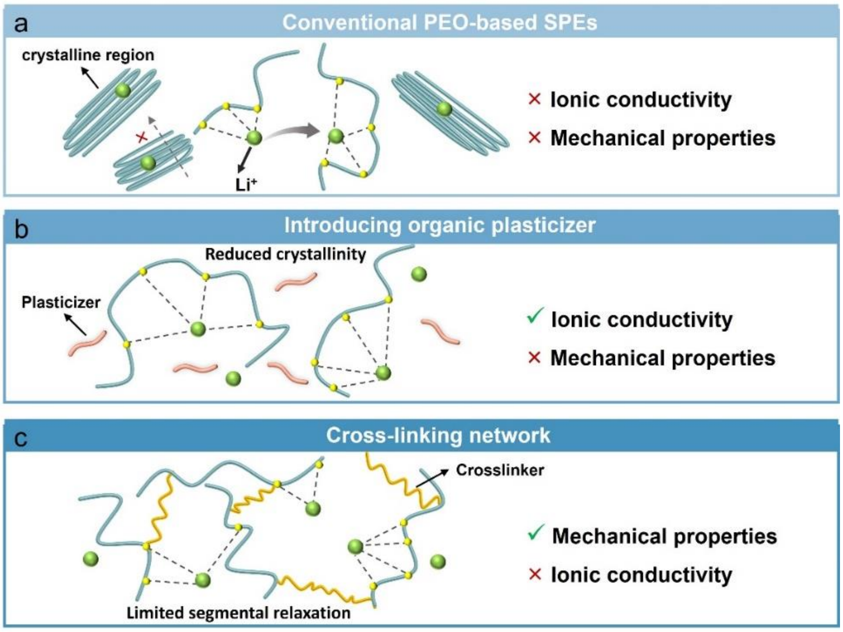

Figure S1. Li$^+$ transport in (a) the conventional PEO-based SPEs, (b) SPEs with organic plasticizer and (c) SPEs with functional crosslinker.

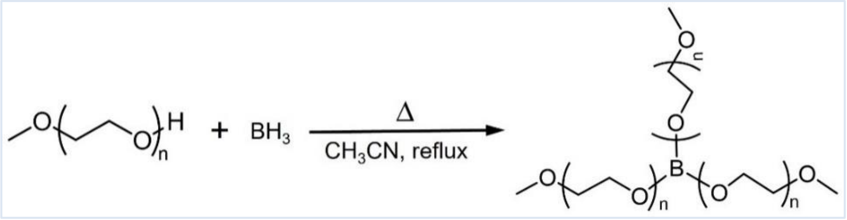

Figure S2. Reaction scheme for the synthesis of MBO-500.

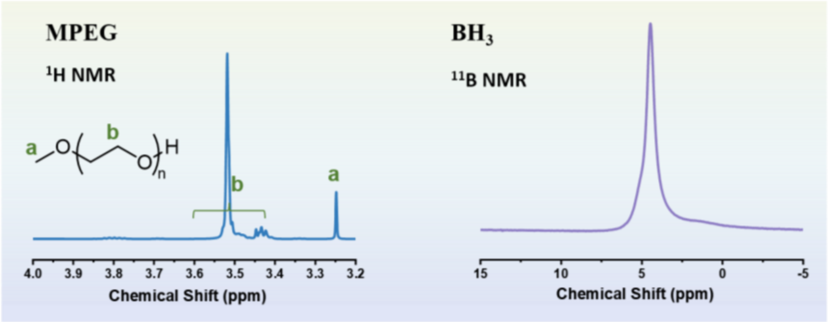

Figure S3. ^{1}H-NMR spectrum of MPEG (Mn=550) and ^{11}B-NMR spectrum of BH_3.

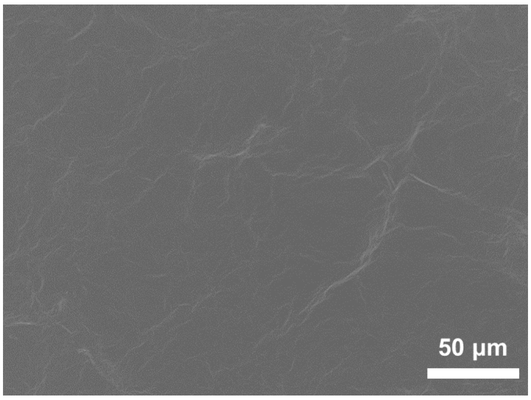

Figure S4. SEM image of the obtained PCPE membrane.

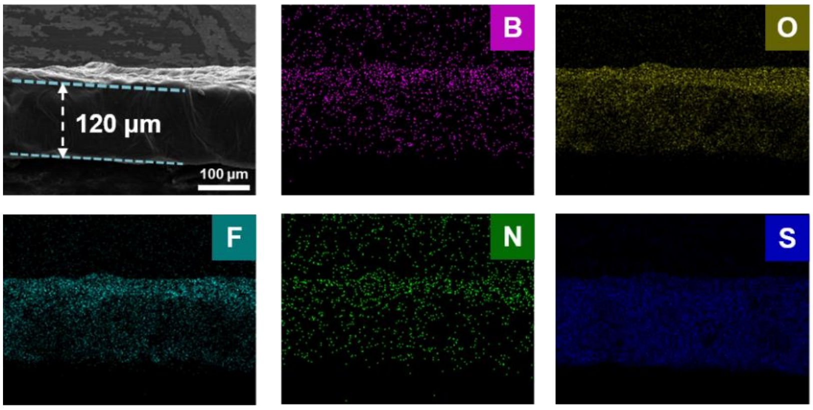

Figure S5. Cross-sectional SEM image of the PCPE and corresponding EDS-mapping (B, O, F, N and S elements) images.

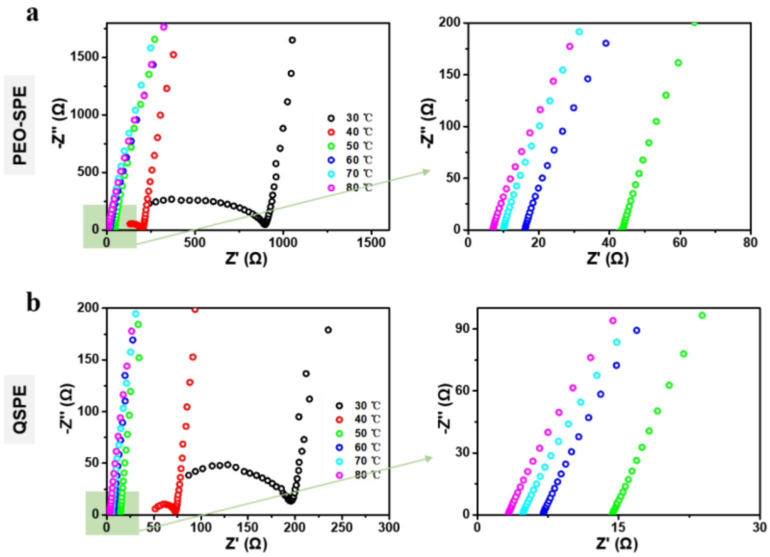

Figure S6. Nyquist plots of (a) PEO-SPE and (b) PCPE-550 at various temperatures

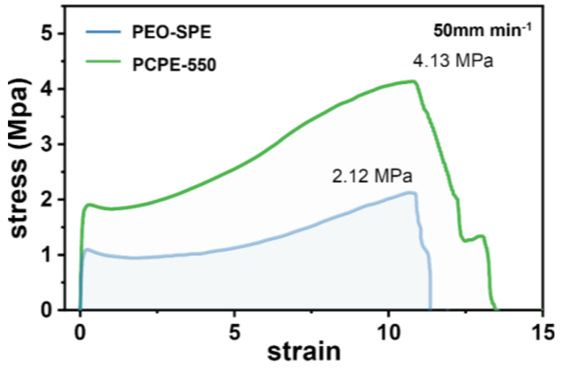

Figure S7. The stress-strain curves of PEO-SPE and PCPE-550 at room-temperature.

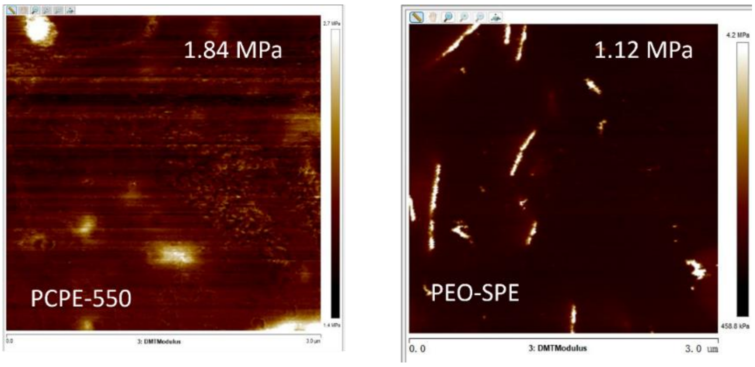

Figure S8. Young's modulus result from AFM test of PCPE-550 and PEO-SPE membranes.

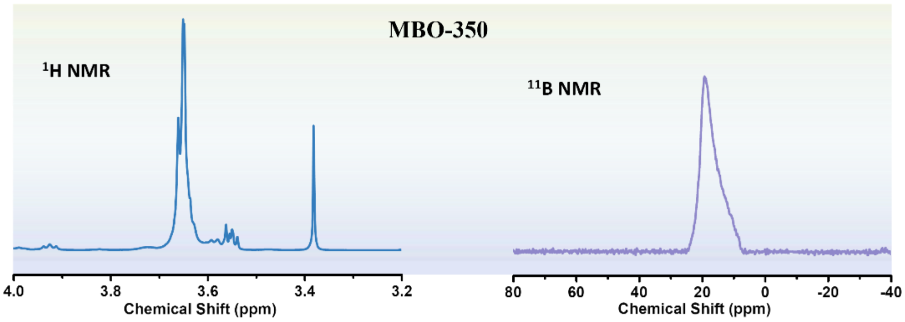

Figure S9. The ^{1}H-NMR and ^{11}B-NMR spectra of MBO-350.

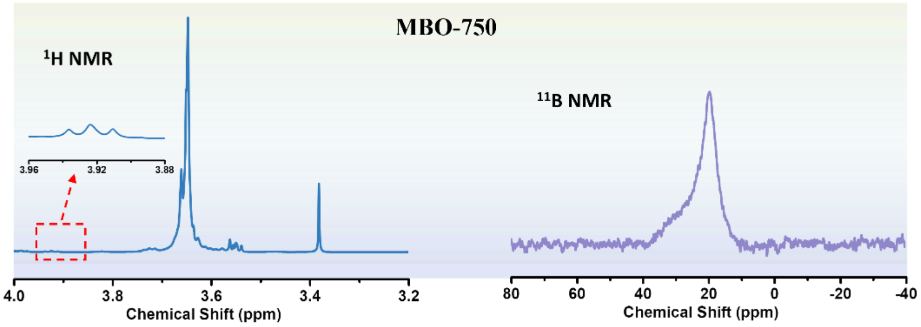

Figure S10. The ^{1}H-NMR and ^{11}B-NMR spectra of MBO-750.

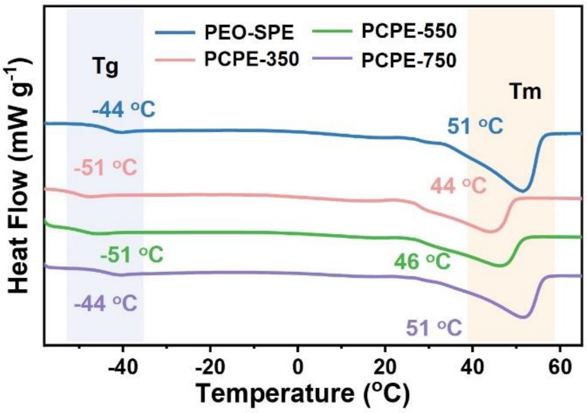

Figure S11. DSC results of PEO-SPE and PCPE using different MBO plasticizers.

Table S1. The melting enthalpy and crystallinity of PEO-SPE and PCPE.

	**Melting enthalpy(J/g)**	**Crystallinity (%)**
PEO-SPE	42.23	30.6
PCPE-350	32.04	24.0
PCPE-550	32.77	24.6
PCPE-750	43.01	32.3

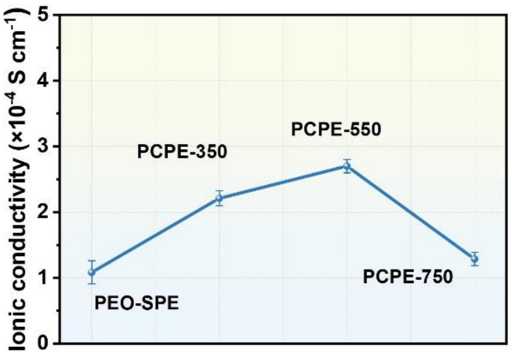

Figure S12. Ionic conductivity of PEO-SPE and PCPE electrolytes using MBO with different molecular weights at 50 °C.

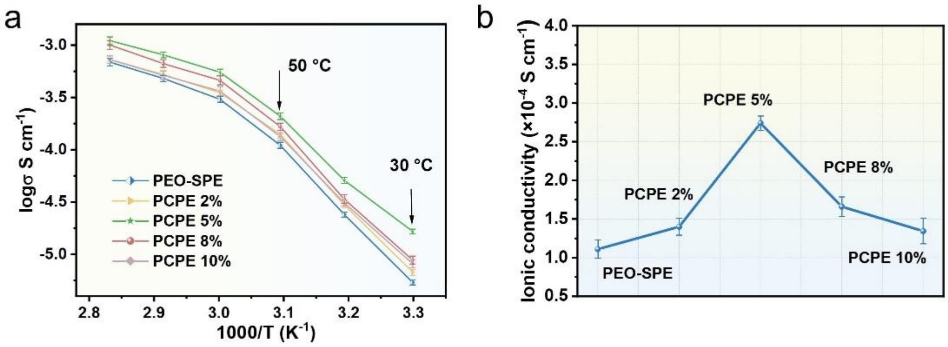

Figure S13. (a) Temperature dependency comparison of ionic conductivity for different electrolytes from 30 to 80 °C and (b) calculated ionic conductivity for different electrolytes at 50 °C.

Figure S13 presents the Li^+ conductivity and stress-strain curves of PCPE with MBO-550 mass content of 2%, 5%, 8%, and 10%. Due to the plasticizing effect and formation of MBO-salt assembly superstructure, the PCPE with MBO-550 content of 5% exhibits the highest ionic conductivity. The ionic conductivity of PCPE with MBO-550 content of 2% is lower than that of 5%, as the low MBO-550 content could not ensure the formation of enough MBO-salt assembly superstructure in PCPE environment. When the content of MBO-550 in PCPE is above 5%, the Li^+ conductivity began to decline. This might be attributed to the excessive physical crosslinking under high MBO-550 content in suparmolecularly extended polymer network, which limits the movement of PEO segments and leads to limited ion migration paths. The higher

MBO-550 content, the higher physical crosslinking in PCPE between MPEG side chains of MBO or MBO-salt assembly superstructure and PEO matrix. Therefore, it can be seen from following Figure S14 that the mechanical properties of the PCPE continue to increase with increasing the content of MBO-550.

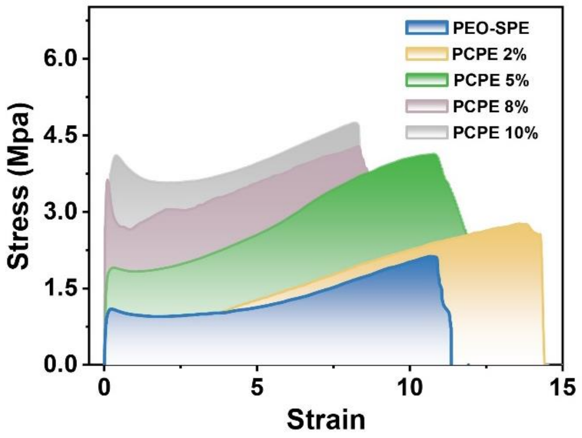

Figure S14. Stress-strain curves of PCPE-550 with different MBO-550 contents.

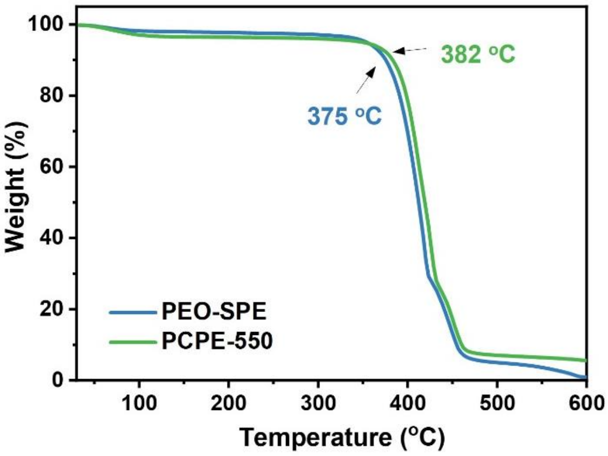

Figure S15. TGA curves of the PEO-SPE and PCPE-550.

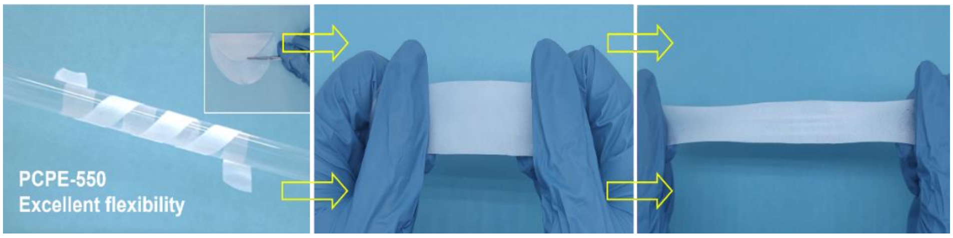

Figure S16. Mechanical flexibility display of the PCPE-550.

Table S2. EIS fitting results of Li/PCPE-550/Li for the transport number test.

**EIS fitting result of Li/PCPE-550/Li before polarization (50 ºC)**				
Element	Definition	Value	Unit	Error (%)
Re	Resistance of electrolyte	32.91	Ω	0.96
Rf	Resistance of the passivating layer at the electrode/electrolyte interface	134.2	Ω	2.47
CPEf-T	Constant phase element of the passivating layer at the electrode/electrolyte interface	1.79E-6	$S\cdot s^n\cdot cm^{-2}$	0.90
CPEf-P		0.80	/	1.56
Rc	Resistances of charge transfer	265.6	Ω	1.14
CPEc-T	Constant phase element of charge transfer	4.27E-6	$S\cdot s^n\cdot cm^{-2}$	2.77
CPEc-P		0.81	/	2.39
χ^2	0.000245			

**EIS fitting result of Li/PCPE-550/Li After polarization (50 ºC)**				
Element	Definition	Value	Unit	Error (%)
Re	Resistance of electrolyte	45.28	Ω	1.42
Rf	Resistance of the passivating layer at the electrode/electrolyte interface	166.0	Ω	1.78
CPEf-T	Constant phase element of the passivating layer at the electrode/electrolyte interface	1.15E-6	$S\cdot s^n\cdot cm^{-2}$	1.19
CPEf-P		0.84	/	1.50
Rc	Resistances of charge transfer	473.1	Ω	2.24
CPEc-T	Constant phase element of charge transfer	4.13E-6	$S\cdot s^n\cdot cm^{-2}$	1.97
CPEc-P		0.81	/	3.06
χ^2	0.000961			

Table S3. Transport number for PCPE-550 electrolyte.

**Parameters**	**PCPE**	**Unit**
Lithium-ion transport number	0.63	-
Polarization voltage (ΔV)	0.01	V
Initial current (I_0)	1.25E-5	A
Steady state current (I_s)	8.3E-6	A
The initial resistance of the electrolyte (Rb_0)	45.07	Ω
Final resistance of the electrolyte (Rb_s)	32.94	Ω
Initial interfacial resistance (R_0)	430.13	Ω
Steady-state interfacial resistance (R_s)	674.27	Ω

Table S4. EIS fitting results of Li/PEO-SPE/Li for the transport number test.

EIS fitting result of Li/PEO-SPE/Li before polarization (50 ºC)				
Element	Definition	Value	Unit	Error (%)
Re	Resistance of electrolyte	48.16	Ω	1.18
Rf	Resistance of the passivating layer at the electrode/electrolyte interface	170.8	Ω	1.69
CPEf-T	Constant phase element of the passivating layer at the electrode/electrolyte interface	2.38E-6	$S{\cdot}s^n{\cdot}cm^{-2}$	1.10
CPEf-P		0.80	/	1.47
Rc	Resistances of charge transfer	1465.0	Ω	1.13
CPEc-T	Constant phase element of charge transfer	2.82E-6	$S{\cdot}s^n{\cdot}cm^{-2}$	0.79
CPEc-P		0.85	/	0.86
χ^2	0.000125			
**EIS fitting result of Li/PEO-SPE/Li After polarization (50 ºC)**				
Element	Definition	Value	Unit	Error (%)
Re	Resistance of electrolyte	47.81	Ω	0.99
Rf	Resistance of the passivating layer at the electrode/electrolyte interface	166.6	Ω	1.60
CPEf-T	Constant phase element of the passivating layer at the electrode/electrolyte interface	2.18E-6	$S{\cdot}s^n{\cdot}cm^{-2}$	1.00
CPEf-P		0.81	/	1.35
Rc	Resistances of charge transfer	1486.0	Ω	1.05
CPEc-T	Constant phase element of charge transfer	2.88E-6	$S{\cdot}s^n{\cdot}cm^{-2}$	0.73
CPEc-P		0.85	/	0.80
χ^2	0.001031			

Table S5. Transport number for PEO-SPE electrolyte.

Parameters	PEO-SPE	Unit
Lithium-ion transport number	0.20	-
Polarization voltage (ΔV)	0.01	V
Initial current (I_0)	5.5E-6	A
Steady state current (I_s)	4.13E-6	A
The initial resistance of the electrolyte (Rb_0)	52.91	Ω
Final resistance of the electrolyte (Rb_s)	52.20	Ω
Initial interfacial resistance (R_0)	1680.00	Ω
Steady-state interfacial resistance (R_s)	1715.90	Ω

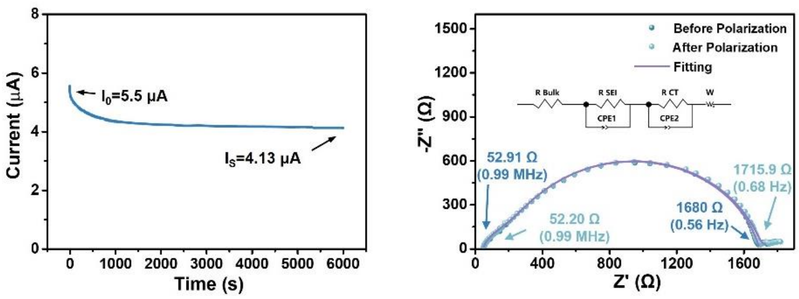

Figure S17. The time-dependent response of the direct-current polarization and the impedance spectra before and after chronoamperometry of PEO-SPE.

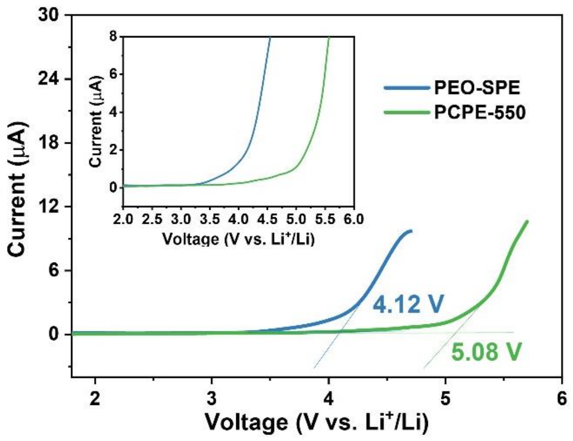

Figure S18. LSV curves of PCPE-550 and PEO-SPE at a scanning rate of 10 μV/s.

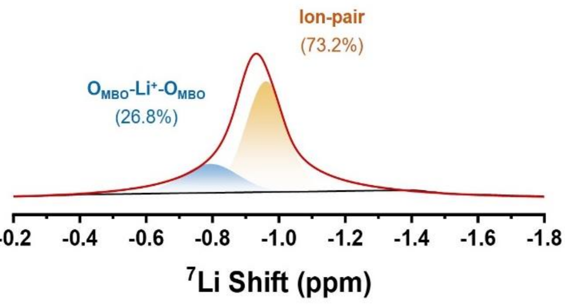

Figure S19. ^{7}Li solid-state NMR spectrum of the MBO with LiTSFI.

Table S6. The calculated coordination numbers of the Li^+/X pairs.

System	Li-O_{PEO}	Li-O_{TFSI^-}	Li-O_{MBO}
PEO-SPE	4.76	0.93	
PCPE-550	4.70	0.80	0.18

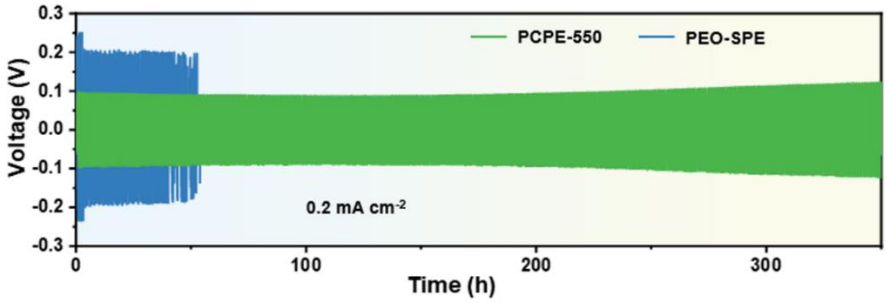

Figure S20. Comparison of cycling performance of Li/Li symmetric cells with the PCPE-550 and PEO-SPE at 0.2 mA cm^{-2}.

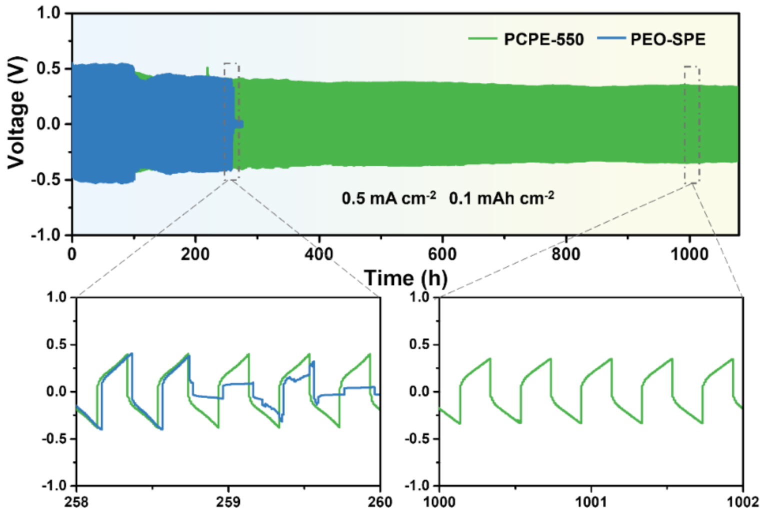

Figure S21. Comparison of cycling performance of Li/Li symmetric cells with the PCPE-550 and PEO-SPE at 0.5 mA cm^{-2}.

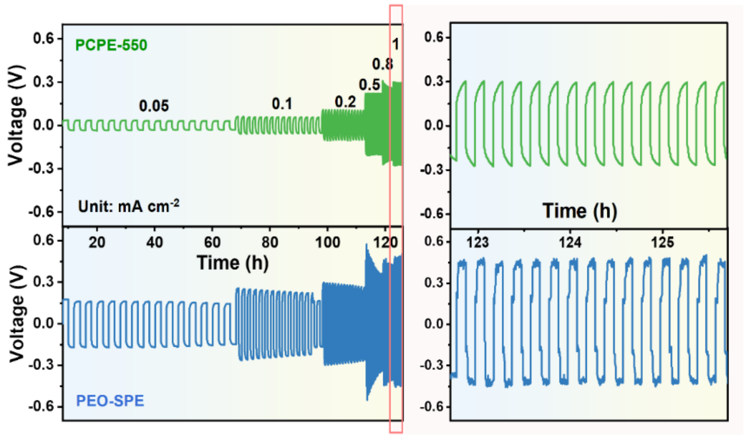

Figure S22. Voltage profiles of repeated Li plating/stripping in symmetric cells with PCPE-550 and PEO-SPE at different current densities.

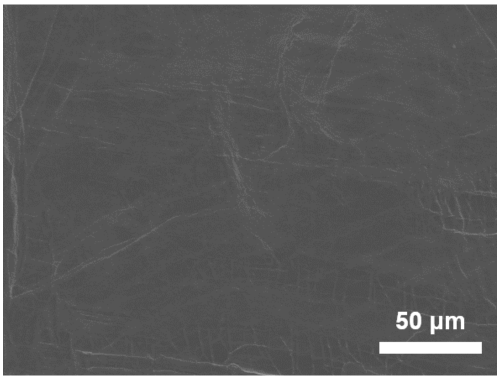

Figure S23. SEM image of the original Li metal anode before cycling.

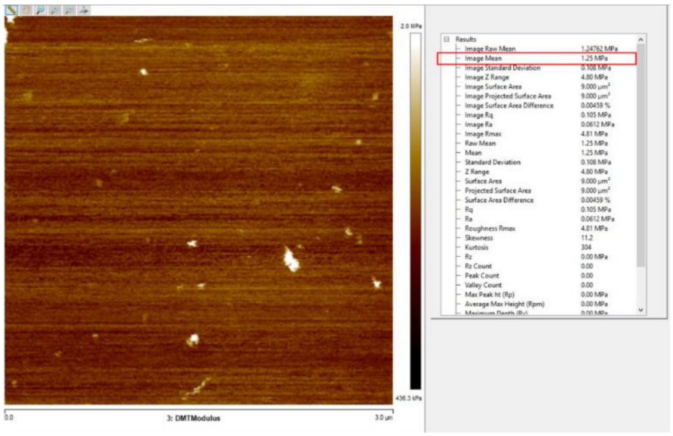

Figure S24. Young's modulus result from AFM test of PCPE-550 membrane after cycling tests.

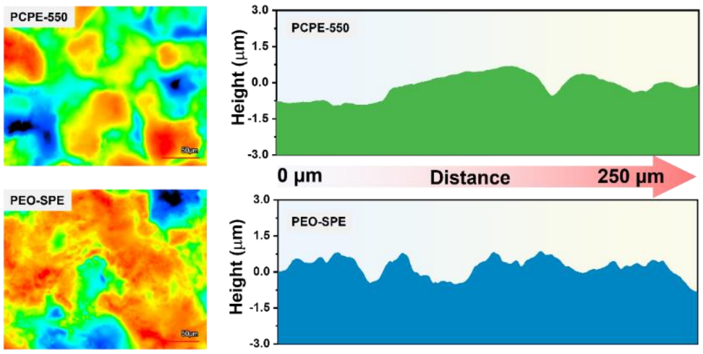

Figure S25. The surface morphology and height images of Li metal anodes disassembled from Li/Li symmetrical cells by 3D CLSM.

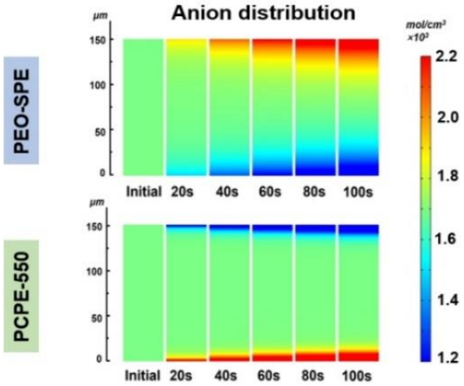

Figure S26. The TFSI^{-} concentration distribution in the PCPE-550 and PEO-SPE electrolytes during the Li plating process.

Table S7. Performance comparison of different polymer electrolytes for all-solid-state lithium batteries.

**Solid-state electrolytes**	**Temperature**	**Ionic conductivity (S cm^{-1})**	**Transport number**	**Ref.**
PCPE-550	50 °C	2.70×10^{-4}	0.63	**This work**
SCE	55 °C	9.1×10^{-5}	0.47	[8]
PEO-Ca-CeO_2	60 °C	1.3×10^{-4}	0.453	[9]
VCOF-SPE	60 °C	2.66×10^{-4}	0.38	[10]
PEO-Bi/HMT MOFs	60 °C	5.86×10^{-4}	0.531	[11]
PEO-SCP	60 °C	2.43×10^{-4}	0.42	[12]
PEO-LLZO	60 °C	4.68×10^{-4}	0.41	[13]
CNF-COF@PEO SPE	60 °C	6.34×10^{-4}	0.81	[14]
PEO-FCN	60 °C	4×10^{-4}	0.43	[15]
AMIC	60 °C	1.78×10^{-4}	0.67	[16]
PEO-LiBTFSI	70 °C	3.6×10^{-4}	0.69	[17]
PEO-in situ PACA	60 °C	1.41×10^{-4}	0.52	[18]
PEO/LiTFSI/TiO_2	50 °C	5.8×10^{-4}	0.36	[19]
hbPPEGMA$_{50}$-s-PS$_{299}$	60 °C	9.1×10^{-5}	0.22	[20]

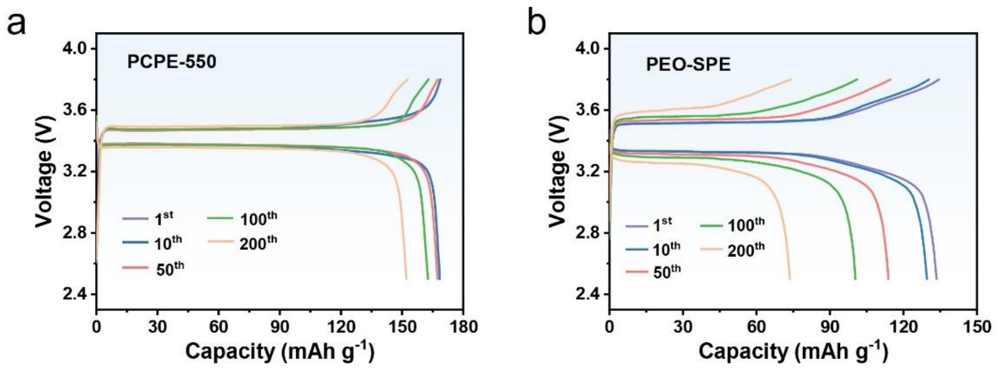

Figure S27. Charge/discharge profiles of different cycles of (a) LFP/PCPE-550/Li and (b) LFP/PEO-SPE/Li cells at 0.2 C, respectively.

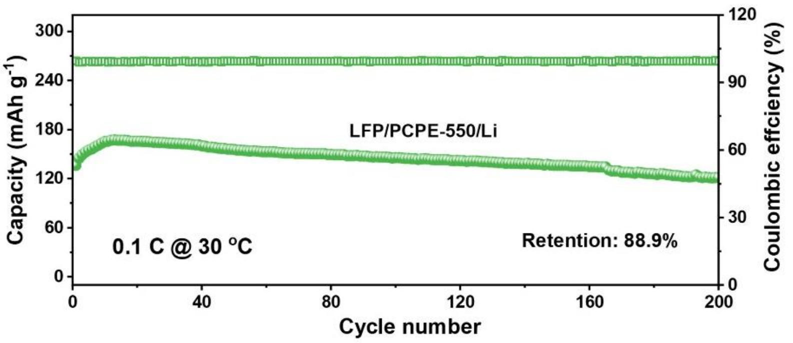

Figure S28. Cycling performance of all-solid-state LFP/PCPE-550/Li cell at 0.1 C at 30 °C.

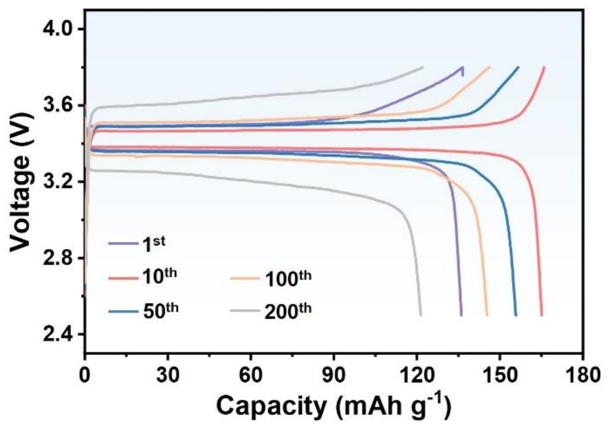

Figure S29. Charge/discharge profiles of different cycles of all-solid-state LFP/PCPE-550/Li cell at 0.1 C at 30 °C.

Table S8. Comparison of electrochemical performance of polymer electrolyte-based all-solid-state lithium batteries (Coin cells).

**Solid-state electrolytes**	**Temperature**	**Cathode material**	**Capacity (mAh g^{-1})**	**Cycle number**	**Capacity retention**	**Ref.**
PCPE-550	50 °C	LFP	167.2 (0.2 C)	200	90.5%	This work
	30 °C		136.5 (0.1 C)	200	88.9%	
VCOF-SPE	60 °C	LFP	142.6 (0.1 C)	150	-	[10]
PEO-LLZO	60 °C	LFP	160.6 (0.2 C)	100	-	[13]
S-NCM /PEO	30 °C	NCM	153.4 (0.05 C)	50	81.1%	[21]
PAAA-PEO	60 °C	LFP	156.7 (0.1 C)	50	98.4%	[22]
SCE	30 °C	LFP	118.6 (0.1 C)	200	93.4%	[8]
PEO-Bi/HMT MOFs	60 °C	LFP	158.6 (0.2 C)	235	91.15%	[11]
AMIC	60 °C	NCM	168.7 (0.1 C)	200	85%	[16]
CNF-COF@PEO SPE	60 °C	LFP	137.2 (0.2 C)	500	97.2%	[14]
PVDF/PEO/PVDF	60 °C	LFP	152 (0.1 C)	125	99.4%	[23]
IPN9-10PPC	90 °C	LFP	152 (0.2 C)	200	92.7%	[24]
PUSPE	60 °C	LFP	160.3 (0.1 C)	200	92.4%	[25]

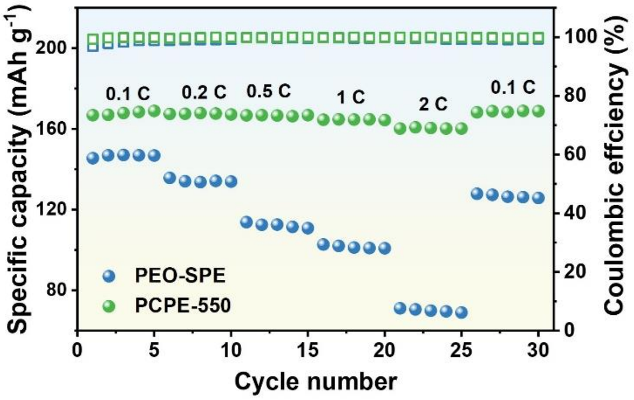

Figure S30. Comparison of the rate capability of all-solid-state LFP/Li batteries using different electrolytes.

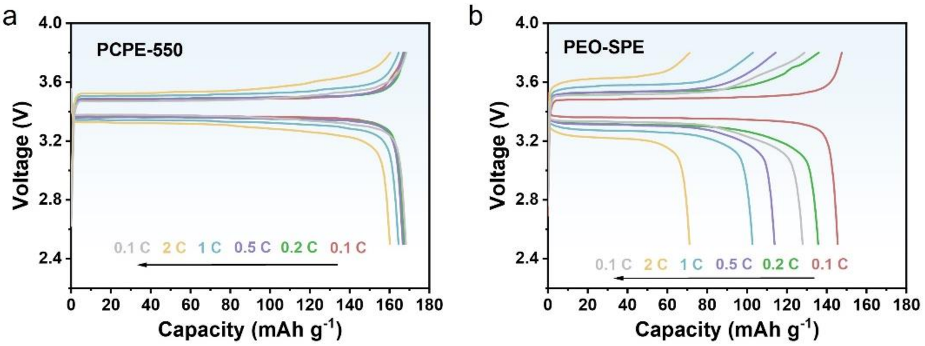

Figure S31. Charge/discharge profiles of (a) LFP/PCPE-550/Li and (b) LFP/PEO-SPE/Li cells at different current densities, respectively.

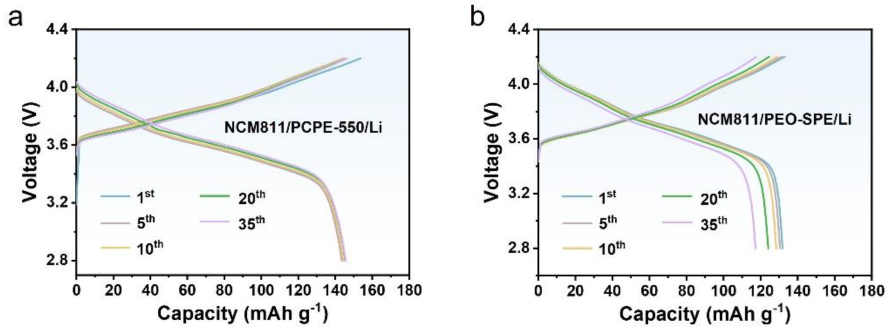

Figure S32. Charge/discharge profiles of NCM811/Li cells using (a) PCPE-550 and (b) PEO-SPE electrolytes at 0.5 C.

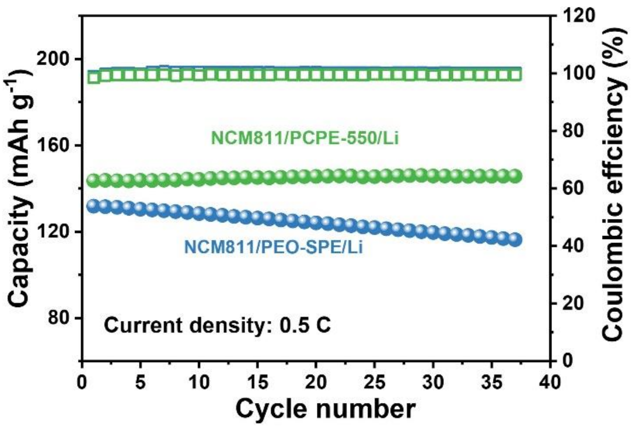

Figure S33. Cycling performance of NCM811/PCPE-550/Li and NCM811/PEO-SPE/Li cells at 0.5 C.

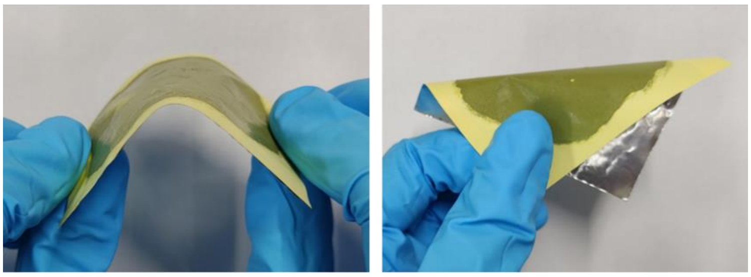

Figure S34. The digital photos of the integrated cathode electrode.

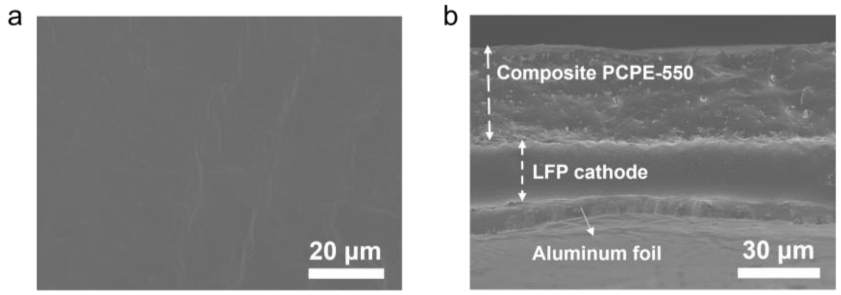

Figure S35. The SEM images of the integrated LFP cathode.

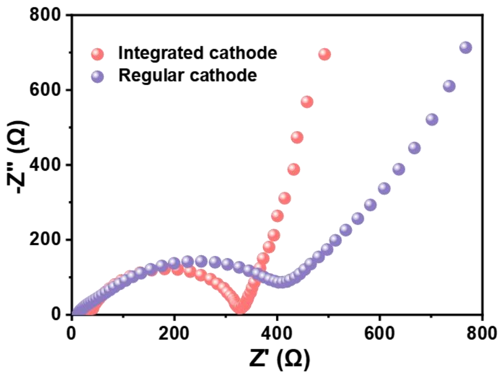

Figure S36. EIS plots of the all-solid-state LFP/Li pouch cells using integrated cathode and regular cathode before cycling.

Table S9. Comparison of electrochemical performance of polymer electrolyte based all-solid-state lithium batteries (Pouch cells).

**Solid-state electrolytes**	**Temperature**	**Mass loading (mg cm^{-2})**	**Cathode material**	**Capacity (mAh g^{-1})**	**Cycle number**	**Capacity retention**	**Ref.**
PCPE-550	50 °C	12.4	LFP	average 159 (0.1 C)	100	89%	**This work**
SPC	30 °C	6.0	LFP	average 123.7 (0.1 C)	50		[26]
FDEO	55 °C	4.0	LFP	150.2 (0.1 C)	1		[27]
OV-LLZTO/PEO	60 °C	2.0	LFP	120 (5 C)	500	75.68%	[28]
VCOF-SPE	60 °C	1.2	LFP	80 (0.1 C)	100		[10]
PEO-FCN	60 °C	1.0	NCM	153 (0.1 C)	30	98.4%	[15]

References

[1] R. Li, H. Hua, X. Yang, J. Tian, Q. Chen, R. Huang, X. Li, P. Zhang, J. Zhao, *Energy Environ. Sci.* **2024**, *17*, 5601-5612.

[2] A.D. Becke, *J. Chem. Phys.* **1993**, *98*, 5648-5652.

[3] D. Wang, H. Liu, F. Liu, G. Ma, J. Yang, X. Gu, M. Zhou, Z. Chen, *Nano Letters* **2021**, *21*, 4757-4764.

[4] C. Bannwarth, E. Caldeweyher, S. Ehlert, A. Hansen, P. Pracht, J. Seibert, S. Spicher, S. Grimme, *WIREs Computational Molecular Science* **2021**, *11*, e1493.

[5] C. Bannwarth, S. Ehlert, S. Grimme, *Journal of Chemical Theory and Computation* **2019**, *15*, 1652-1671.

[6] T. Lu, F. Chen, *J. Comput. Chem.* **2012**, *33*, 580-592.

[7] W. Humphrey, A. Dalke, K. Schulten, *Journal of Molecular Graphics* **1996**, *14*, 33-38.

[8] H. Huo, Y. Chen, J. Luo, X. Yang, X. Guo, X. Sun, *Adv. Energy Mater.* **2019**, *9*, 1804004.

[9] H. Chen, D. Adekoya, L. Hencz, J. Ma, S. Chen, C. Yan, H. Zhao, G. Cui, S. Zhang, *Adv. Energy Mater.* **2020**, *10*, 2000049.

[10] J. Yang, C. Lin, Y. Wang, Y. Xu, D.T. Pham, X. Meng, K.V. Tran, S. Cao, N. Kardjilov, A. Hilger, J.D. Epping, I. Manke, A. Thomas, Y. Lu, *J. Mater. Chem. A* **2024**, *12*, 1694-1702.

[11] J. Xu, G. Ma, N. Wang, S. Zhao, J. Zhou, *Small* **2022**, *18*, 2204163.

[12] Z. Chen, J. Li, F. Qiu, C. Lu, J. Zhu, X. Zhuang, *J. Mater. Chem. A* **2022**, *10*,

14849-14856.

[13] Q. Shen, D. Jiang, S. Cao, X. Lu, C. Mao, X. Dai, F. Chen, *ACS Appl. Mater. Interfaces* **2023**, *15*, 38759-38768.

[14] X. Yang, L. Fang, J. Li, C. Liu, L. Zhong, F. Yang, X. Wang, Z. Zhang, D. Yu, *Angew. Chem. Int. Ed.* **2024**, *63*, e202401957.

[15] S. Liu, J. Shen, Z. Wang, W. Tian, X. Han, Z. Chen, H. Pan, L. Wang, D. Bian, C. Yang, S. Zhu, *J. Mater. Chem. A* **2024**, *12*, 256-266.

[16] C. Wang, X. Zhao, D. Li, C. Yan, Q. Zhang, L.-Z. Fan, *Angew. Chem. Int. Ed.* **2024**, *63*, e202317856.

[17] L. Qiao, S. Rodriguez Peña, M. Martínez-Ibañez, A. Santiago, I. Aldalur, E. Lobato, E. Sanchez-Diez, Y. Zhang, H. Manzano, H. Zhu, M. Forsyth, M. Armand, J. Carrasco, H. Zhang, *J. Am. Chem. Soc.* **2022**, *144*, 9806-9816.

[18] Z. Hu, F. Ji, Y. Zhang, W. Guo, X. Jing, W. Bao, J. Qin, S. Huo, S. Li, Y. Zhang, W. Fan, H. Cheng, *Chem. Eng. J.* **2023**, *468*, 143857.

[19] B. Luo, W. Wang, Q. Wang, W. Ji, G. Yu, Z. Liu, Z. Zhao, X. Wang, S. Wang, J. Zhang, *Chem. Eng. J.* **2023**, *460*, 141329.

[20] Y. Chen, Y. Shi, Y. Liang, H. Dong, F. Hao, A. Wang, Y. Zhu, X. Cui, Y. Yao, *ACS Appl. Energy Mater.* **2019**, *2*, 1608-1615.

[21] M. Zuo, Z. Bi, X. Guo, *Chem. Eng. J.* **2023**, *463*, 142463.

[22] P. Chen, Q. Zeng, Q. Li, R. Zhao, Z. Li, X. Wen, W. Wen, Y. Liu, A. Chen, Z. Li, X. Liu, L. Zhang, *Chem. Eng. J.* **2022**, *427*, 132025.

[23] Q. Ye, H. Liang, S. Wang, C. Cui, C. Zeng, T. Zhai, H. Li, *J. Energy Chem.*

2022, *70*, 356-362.

[24] Y. Zheng, X. Li, C.Y. Li, *Energy Storage Mater.* **2020**, *29*, 42-51.

[25] Y. Huang, Z. Shi, H. Wang, J. Wang, Z. Xue, *Energy Storage Mater.* **2022**, *51*, 1-10.

[26] H. Zhou, Y. Ou, S. Yan, J. Xie, P. Zhou, L. Wan, Z.-A. Xu, F. Liu, W. Zhang, Y. Xia, K. Liu, *Angew. Chem. Int. Ed.* **2023**, *62*, e202306948.

[27] Y. Lin, T. Wang, L. Zhang, X. Peng, B. Huang, M. Wu, T. Zhao, *Nano Energy* **2022**, *99*, 107395.

[28] Y. Fu, K. Yang, S. Xue, W. Li, S. Chen, Y. Song, Z. Song, W. Zhao, Y. Zhao, F. Pan, L. Yang, X. Sun, *Adv. Funct. Mater.* **2023**, *33*, 2210845.
